# Single-cell and spatial omics of metabolic-immune ecosystems in prostate cancer: from androgen signaling to therapy resistance

**DOI:** 10.3389/fimmu.2026.1899588

**Published:** 2026-08-06

**Authors:** Ruiang Wang, Yifan Li, Xiaoxiang Wang

**Affiliations:** 1 The First Clinical Medical College, Faculty of Medicine, Yangzhou University, Yangzhou, China; 2 Department of Urology, The Affiliated Hospital of Yangzhou University, Yangzhou University, Yangzhou, China

**Keywords:** androgen receptor, castration resistance, metabolism, neuroendocrine prostate cancer, prostate cancer, single-cell RNA sequencing, spatial transcriptomics, therapy resistance

## Abstract

Prostate cancer is a hormone-driven malignancy, but its clinical behavior cannot be explained by androgen receptor (AR) signaling alone. Single-cell and spatial omics are redefining prostate cancer as an evolving metabolic-immune ecosystem in which malignant epithelial states, stromal niches, myeloid programs, T-cell exclusion, vascular remodeling, and treatment-imposed selection pressures jointly shape progression and resistance. These technologies have resolved the normal prostate epithelial hierarchy, pre-existing castration-resistant-like cells, basal-like, club-like, and hillock-like programs, neuroendocrine transformation states, immunosuppressive macrophage populations, fibroblast states, and spatial neighborhoods that are difficult to detect by bulk sequencing. Treatment-associated changes have been characterized most directly in androgen-axis and immune contexts, whereas evidence related to taxanes, DNA damage response (DDR)-targeted therapy, and radioligand therapy remains emerging or hypothesis-generating. This review synthesizes single-cell and spatial omics evidence for metabolic-immune ecosystems in prostate cancer, emphasizing the transition from androgen dependence to castration resistance, lineage plasticity, metastatic niche adaptation, and therapy resistance. We argue that the next translational step is not simply to catalog additional cell types, but to connect longitudinal cell-state maps, spatially resolved metabolic dependencies, immune-neighborhood biomarkers, and mechanism-matched combination therapies.

## 1 Introduction

Prostate cancer has traditionally been understood through an endocrine lens: malignant growth depends on androgen receptor (AR) activity, androgen deprivation therapy (ADT) can induce tumor regression, and most lethal tumors ultimately reactivate AR signaling or escape AR dependence. This framework remains clinically useful, as next-generation AR signaling inhibitors (ARSIs), taxanes, poly(ADP-ribose) polymerase (PARP) inhibitors for selected DNA repair-defective tumors, and prostate-specific membrane antigen (PSMA)-targeted radioligand therapy have improved outcomes (1, 2). However, AR-centered models are incomplete. Some tumors contain castration-resistant-like epithelial cells before treatment (3); some recurrences depend on chromatin and lineage programs rather than simple AR restoration (4, 5); and many advanced tumors display immune suppression, stromal remodeling, and metabolic reprogramming that influence treatment response (6, 7).

Bulk genomics has identified recurrent alterations such as ETS fusions, SPOP mutations, CHD1 deletion, PTEN loss, FOXA1 mutation, TP53/RB1 loss, DNA repair defects, and AR structural variation (8, 9). However, bulk-tissue analysis averages malignant clones together with benign epithelium, fibroblasts, endothelial cells, macrophages, lymphocytes, and bone-niche cells. It can identify pathways, but often cannot determine which cell state carries a pathway, whether that state existed at low abundance before treatment, whether it is protected by extracellular matrix or bone marrow niches, or whether it depends on local nutrient and oxygen gradients. Single-cell RNA sequencing (scRNA-seq), single-cell assay for transposase-accessible chromatin using sequencing (scATAC-seq), spatial transcriptomics, spatial proteomics, and multiplex imaging allow these questions to be asked directly (10, 11).

The central conceptual shift is from “tumor cells plus background” to “ecosystem.” In this view, malignant epithelial cells are selected within gradients of androgen deprivation, inflammatory signaling, extracellular-matrix stiffness, hypoxia, lipid availability, antigen presentation, myeloid suppression, and treatment-induced tissue damage. Conversely, tumors can influence surrounding stromal and immune cells through cytokines, chemokines, growth factors, metabolic competition, and antigen-dependent or antigen-independent immune evasion. Prostate cancer is especially suited to ecosystem models because its evolution often unfolds over years, is shaped by sequential therapies, and commonly involves bone metastasis, a specialized metabolic and immune organ (12, 13). Therapy resistance can therefore be conceptualized as an organizational phenotype: a resistant clone survives because its transcriptional and epigenetic state is compatible with a microenvironment that permits survival.

The concept of a metabolic-immune ecosystem emphasizes that prostate cancer progression is shaped by two closely connected dimensions. The metabolic state influences what tumor cells can synthesize, how they manage oxidative stress, and whether they can survive androgen deprivation, DNA damage, ferroptotic pressure, or nutrient limitation (14, 15). The immune state, in turn, determines whether malignant cells are ignored, eliminated, functionally restrained, spatially excluded, or reshaped by myeloid and stromal cells (16, 17). These processes rarely operate in isolation. Lipid-rich or hypoxic regions often coincide with immunosuppressive myeloid phenotypes; cytokine-enriched inflammatory niches may promote lineage plasticity; extracellular-matrix barriers can restrict both drug penetration and immune-cell trafficking; and treatment-induced cell death may stimulate antitumor immunity while simultaneously triggering suppressive repair programs. Prostate tumors are therefore better understood not as uniform lesions, but as collections of local metabolic and immune niches, each governed by distinct survival pressures.

This review focuses on single-cell and spatial omics studies that connect androgen receptor biology, metabolic context, and immune organization in prostate cancer. We first summarize the rationale, strengths, and limitations of these platforms. We then discuss malignant epithelial states, androgen signaling, metabolic reprogramming, immune and stromal neighborhoods, and treatment-associated resistance trajectories. Finally, we highlight translational opportunities for biomarker development and rational combination therapy. Rather than cataloging every cell type identified by omics studies, this review emphasizes how cellular states and spatial neighborhoods interact to generate clinically meaningful therapy resistance.

Literature identification followed a structured narrative review approach. PubMed was last searched on 17 July 2026 using combinations of “prostate cancer” with “single-cell,” “spatial transcriptomics,” “spatial proteomics,” “androgen receptor,” “metabolism,” “immune microenvironment,” “lineage plasticity,” and “therapy resistance.” Reference lists from relevant reviews and primary studies were also screened. Priority was given to human single-cell or spatial studies, longitudinal or treatment-linked cohorts, mechanistic studies that experimentally tested inferred interactions, and pivotal clinical trials relevant to translation. Because this is a narrative rather than systematic review, no formal meta-analysis or risk-of-bias assessment was performed. For the core single-cell and spatial evidence synthesis, studies were prioritized for inclusion if they used single-cell or spatial platforms in human prostate tissue, reported a defined cohort with disease stage and treatment context, or provided mechanistic or functional validation of a computationally inferred finding. Conference abstracts without a peer-reviewed full text, studies with no human-tissue component, and duplicate reports of the same dataset were excluded from the core evidence set. Mechanistic, animal, platform, and clinical-trial studies without a human-tissue component were retained only when they provided essential methodological, functional, or translational context.

## 2 Single-cell and spatial omics: platforms, integration, and interpretation

Because this review integrates single-cell and spatial data with bulk transcriptomics, metabolomics, mechanistic and animal studies, and clinical trials, we use four conceptual evidence categories to interpret major claims. We distinguish four levels: (i) descriptive association, in which a cell state, pathway score, or spatial co-occurrence is observed without further testing; (ii) computational prediction, in which ligand-receptor, trajectory, or integrative algorithms infer a relationship that has not been experimentally probed; (iii) functional validation, in which the inferred relationship has been tested by genetic, pharmacological, co-culture, or *in vivo* perturbation, or by an orthogonal protein-level assay; and (iv) clinical evidence, in which a finding is supported by outcome data from a defined, appropriately treated patient cohort or trial. **Table 1** summarizes the platform, cohort and sample characteristics, study design, spatial resolution, validation status, and principal limitation of the single-cell and spatial studies most central to this review, organized by anatomical compartment (primary tumor, mixed primary-recurrent-metastatic cohorts, bone metastasis, and lymph-node/visceral metastasis). Unless stated otherwise, claims in the text should be read as level (i) or (ii) evidence; findings with functional or clinical support are described as such and summarized in **Table 1**.

**Table 1:** Principal single-cell and spatial studies in prostate cancer.

Study	Cohort and clinical context	Platform and study design	Principal finding	Validation and major limitation
Primary tumors				
Hirz et al., 2023 (6)	19 patients + 5 controls; primary tumors	scRNA-seq + ST (spot-level); cross-sectional	Suppressive myeloid cells and exhausted T cells characterize an immunosuppressive TME.	Computational interaction inference only; no functional validation.
Kiviaho et al., 2024 (10)	120 patients; primary tumors, pooled stages	scRNA-seq + ST (spot-level); cross-sectional	Club-like cells co-localize with PMN-MDSCs and may relate to ADT resistance.	Spatial association only; causality untested.
Tuong et al., 2021 (16)	10 paired patients; primary tumors	scRNA-seq (non-spatial); cross-sectional	MAC-MT macrophages were associated with better disease-free survival.	Validated in independent cohorts; no spatial or causal evidence.
Karthaus et al., 2020 (18)	Mouse and human primary tissue	scRNA-seq (non-spatial) + organoid/*in vivo* models	Stem-like and differentiated luminal cells can both support regeneration.	Strong functional models, but core evidence was murine.
Andersen et al., 2024 (19)	37 patients/176 samples; primary tumors	ST (spot-level) + proteomics + methylomics; cross-sectional	SFRP4 RNA and protein levels were discordant despite links to aggressiveness.	Multi-omic validation; small spatial cohort and no response analysis.
Wu et al., 2025 (20)	23 prostate samples (tumor and non-tumor); IF validation in five patients	scRNA-seq + ST (spot-level); cross-sectional	FAP+ fibroblast-SPP1+ macrophage crosstalk was inferred.	IHC/IF localization; no perturbation test.
Takahara et al., 2025 (21)	6 intraductal carcinoma cases	ST (spot-level) + CNV; cross-sectional	Intraductal carcinoma showed distinct transcriptomic and genomic features.	Histologic cross-check; preliminary sample size.
Cheng Y et al., 2025 (22) (corrected in (23))	10 patients; age-stratified primary tumors	scRNA-seq + ST (spot-level); cross-sectional	AR programs and macrophage/CAF composition differed by age.	Multiplex IF and CAF assays; small discovery cohort. A published correction amended cohort labels and one duplicated image.
Primary, recurrent, and metastatic disease				
Cheng Q et al., 2022 (3)	Primary, recurrent, and metastatic samples; ADT context	scRNA-seq (non-spatial); cross-sectional	CRPC-like and NE-like cells were detectable before ADT.	IHC/public-cohort support; no longitudinal proof.
Taavitsainen et al., 2021 (24)	Treatment-response model systems	scATAC-seq + scRNA-seq (non-spatial); *in vitro* longitudinal	Pre-existing or persistent states may predict treatment response.	Patient correlation, but main evidence was preclinical.
Zaidi et al., 2024 (25)	21 biopsies + 131-sample TMA; mixed stages/sites	scRNA-seq (non-spatial) + TMA; cross-sectional	Antigen expression varied across lineage-plasticity states.	Protein validation; no within-patient evolution data.
Shen et al., 2025 (26)	70 samples from 34 public datasets; NEPC	Single-cell meta-analysis (non-spatial)	NEP100 classified four NEPC subtypes.	*In vitro* support; heterogeneous datasets and annotations.
Lyu et al., 2025 (27)	43 biopsies across localized, hormone-sensitive metastatic, and castration-resistant metastatic disease	scRNA-seq (non-spatial) + mouse models + clinical trial	SPP1-high macrophages contributed to ICI resistance; A2AR inhibition showed promise.	Multi-tier validation; no spatial data and biomarker analysis underpowered.
Bone metastases				
Kfoury et al., 2021 (12)	9 bone-metastasis patients + 7 controls	scRNA-seq (non-spatial) + mouse model; cross-sectional human data	CCL20-CCR6 signaling promoted an immunosuppressive bone niche.	*In vivo* validation was murine; human data were non-spatial.
Ye et al., 2023 (13)	Secondary human data + mouse models; bone metastasis	Single-cell reanalysis (non-spatial) + multi-omics	Myeloid-like hybrid cells promoted bone-metastatic progression.	Extensive mouse validation; small secondary human dataset.
Mei et al., 2025 (28)	Integrated datasets including 103 prostate cancer samples; human bone-metastasis specimens n=4 and distal samples n=4	scRNA-seq + ST (spot-level) + multiplex IF	SPP1+/TREM2+ macrophages were enriched and linked to worse PFS.	Human spatial association plus imaging; SPP1 blockade evidence was derived from a mouse model.
Corn et al., 2025 (29)	24 paired patients; only 3 interpretable pairs	Multiplex IF (single-cell protein imaging); longitudinal	Radium-223 produced heterogeneous tumor and T-cell changes.	True paired design, but extremely small evaluable sample.
Mouse models with human primary and metastatic validation				
Pakula et al., 2024 (30)	38 mice; human primary and bone-metastasis validation; bulk validation in 1,239 tumors	Mouse scRNA-seq + human single-cell/bulk validation (non-spatial)	Periostin-linked stroma predicted progression beyond Gleason score.	Cross-species support; core discovery was murine.
Lymph-node and visceral metastases				
Xin et al., 2023 (31) (corrected in (32))	4 primary/lymph-node-metastasis prostate cancer tissue samples; 32,766 cells	scRNA-seq (non-spatial) + targeted experimental validation; cross-sectional	Lymph-node metastasis was associated with immunosuppressive epithelial, immune, and fibroblast states.	Dedicated lymph-node study, but the cohort was very small and no spatial assay was used. A published correction amended Figure 3.
Evidence gap	Liver and lung metastases	No dedicated peer-reviewed human single-cell or spatial study identified	Direct organ-specific evidence remains limited.	Interpretations are extrapolated from primary, lymph-node, bone, or mixed-site studies.

A2AR, adenosine A2A receptor; ADT, androgen-deprivation therapy; AR, androgen receptor; CAF, cancer-associated fibroblast; CNV, copy-number variation; CRPC, castration-resistant prostate cancer; ICI, immune-checkpoint inhibitor; IF, immunofluorescence; IHC, immunohistochemistry; MAC-MT, study-defined macrophage state enriched in malignant tissue; NE, neuroendocrine; NEPC, neuroendocrine prostate cancer; PFS, progression-free survival; PMN-MDSC, polymorphonuclear myeloid-derived suppressor cell; scATAC-seq, single-cell assay for transposase-accessible chromatin using sequencing; scRNA-seq, single-cell RNA sequencing; ST, spatial transcriptomics; TMA, tissue microarray; TME, tumor microenvironment.

### 2.1 Single-cell and spatial platforms

Single-cell omics decomposes tumors into cellular-level measurements, whereas spatial omics places those measurements back into tissue geography. Droplet-based single-cell RNA sequencing enables high-throughput cell profiling (33). Spatial reconstruction methods help restore tissue organization from dissociated data (34), while cross-tissue mouse single-cell atlases provide comparative references for cell annotation (35). In prostate cancer, single-cell RNA sequencing has been used to define normal epithelial and stromal anatomy, primary tumor heterogeneity, metastatic niches, treatment-resistant disease, neuroendocrine transformation, and immune suppression (18, 36). Matched scATAC-seq and scRNA-seq analyses add an epigenetic layer by identifying pre-existing persistent cell states that survive treatment and expand during recurrence (24).

**Figure 1:**
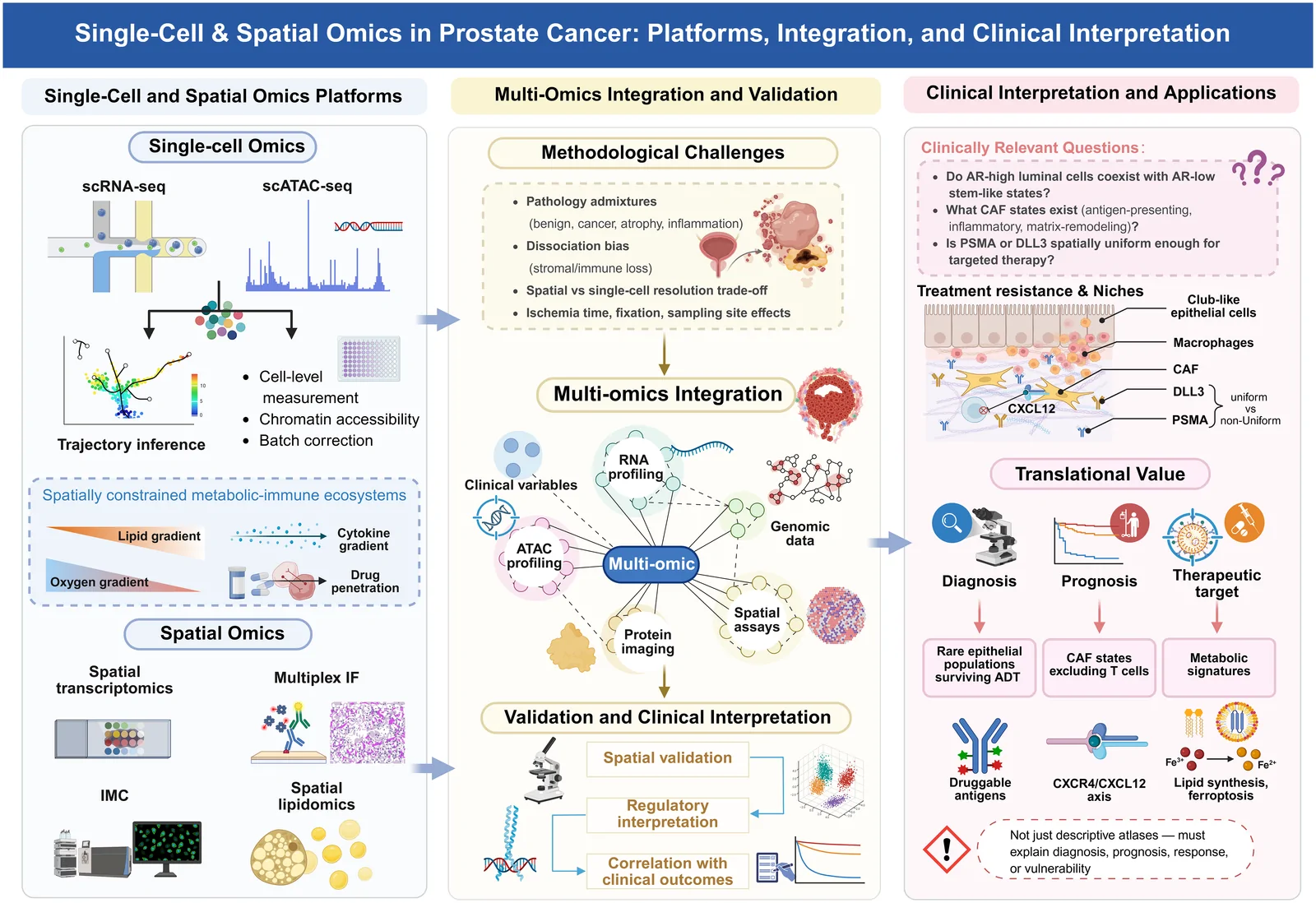
Multi-omics platforms, integration, methodological challenges, and clinical applications in prostate cancer. This schematic illustrates how single-cell and spatial omics platforms can be integrated to characterize prostate cancer heterogeneity and treatment resistance. Single-cell approaches, including scRNA-seq and scATAC-seq, define tumor and microenvironmental cell states, developmental trajectories, chromatin accessibility, and cell-level transcriptional programs. Spatial technologies, including spatial transcriptomics, multiplex immunofluorescence, imaging mass cytometry, and spatial lipidomics, preserve tissue architecture and reveal spatially organized metabolic-immune gradients such as lipid availability, oxygen tension, cytokine distribution, and drug penetration. Multi-omics integration combines RNA, chromatin, protein imaging, spatial assays, genomic data, and clinical variables, while accounting for methodological challenges such as tissue admixture, dissociation bias, spatial-resolution limitations, ischemia time, fixation, and sampling-site effects. Integrated datasets require spatial validation, regulatory interpretation, and correlation with clinical outcomes. Clinically, these approaches may help identify treatment-resistant niches, AR-low or stem-like epithelial states, macrophage-CAF interactions, DLL3-associated regions, heterogeneous PSMA expression, metabolic vulnerabilities, and CXCR4/CXCL12-related immune exclusion. Rather than serving only as descriptive atlases, single-cell and spatial omics should be used to explain mechanisms of prognosis, therapeutic response, and vulnerability in prostate cancer.

Spatial transcriptomics and spatial proteomics address the core limitation of dissociation-based methods: loss of positional information. Early spatial transcriptomic methods demonstrated gene-expression measurement directly on tissue sections (37). Recent prostate cancer studies have used spatial RNA analysis and computational modeling to characterize macrophage-rich neighborhoods and heterogeneous metabolic gene-expression patterns (28, 38). These technologies are particularly suited to metabolic-immune ecosystems because both metabolism and immunity are spatially constrained. Oxygen, lipids, cytokines, antigen presentation, and drug penetration form local gradients associated with specific cellular states (**Figure 1**).

### 2.2 Integration, validation, and interpretation

The field still faces several methodological challenges. First, prostate tumors are often admixed with benign glands, low-grade cancer, high-grade cancer, atrophy, inflammation, and stromal hyperplasia, so cell annotations must be anchored to pathological sampling. Second, tissue dissociation may underrepresent fragile stromal and immune populations, whereas spatial assays may lack single-cell resolution. Third, AR signaling, metabolic pathways, interferon programs, and hypoxia signatures can be affected by ischemia time, fixation, treatment interval, and sampling site. Fourth, primary tumors, lymph-node metastases, bone metastases, and visceral metastases are not interchangeable ecosystems. Finally, many studies remain cross-sectional, whereas therapy resistance is inherently longitudinal. Accordingly, future studies should prioritize baseline and on-treatment biopsies, integrate RNA and chromatin measurements, validate spatial localization, and correlate cell states with clinical outcomes.

Multi-omic integration is becoming a central framework for this field. RNA profiling defines expression states, ATAC profiling infers regulatory accessibility, genomic data identify clonal structure, spatial assays assign neighborhood context, protein imaging verifies surface markers, and clinical variables determine whether a state is clinically meaningful. In prostate cancer, transcriptional assays can identify AR activity and splice-variant-associated resistance (39, 40), whereas chromatin accessibility, copy-number structure, metabolic flux, and stromal protection require corresponding epigenomic, genomic, metabolic, and spatial measurements. Similarly, T-cell exhaustion markers suggest immune dysfunction, but spatial distance from tumor cells, proximity to macrophages, antigen-presentation defects, and cancer-associated fibroblast (CAF) barriers determine whether T cells can act. A high-quality prostate cancer atlas therefore needs to connect layers rather than remain a single-modality cluster directory.

The most convincing integrated studies combine single-cell discovery with spatial validation and regulatory interpretation. A prostate cancer review and a general scATAC-seq methods study emphasize that dissociation transcriptomics, chromatin accessibility, and high-throughput tissue imaging are complementary rather than interchangeable tools (41, 42). Multiplex imaging can further test whether inferred cell states occupy biologically plausible tissue neighborhoods (43).

Despite these limitations, single-cell and spatial omics have changed the questions that prostate cancer studies can ask. Instead of asking only whether a tumor is “AR positive” or “immune cold”, investigators can ask whether AR-high luminal cells coexist with AR-low stem-like states; whether macrophages concentrate near club-like epithelial cells; whether CAFs adopt antigen-presenting, inflammatory, or matrix-remodeling states; whether PSMA or DLL3 expression is spatially uniform enough for targeted therapy; and whether a metabolic program is carried by tumor cells, fibroblasts, myeloid cells, or bone-niche cells.

Interpretation must remain clinically oriented. Cell clusters are useful only when they explain diagnosis, prognosis, treatment response, or targetable vulnerability. For example, rare epithelial populations become clinically important if they survive ADT, express druggable surface antigens, lose PSMA, or occupy macrophage-enriched niches associated with checkpoint resistance (3, 27). CAF states are translationally relevant when they are associated with mitochondrial metabolism, invasive progression, or treatment resistance (30, 44). Metabolic signatures are valuable if they identify patients who may benefit from targeting lipid synthesis, cholesterol metabolism, oxidative stress, or ferroptosis (45, 46). This constraint helps prevent single-cell studies from becoming descriptive atlases without therapeutic consequence.

## 3 Androgen-regulated epithelial and metabolic states

### 3.1 Androgen signaling as an ecosystem organizer

AR signaling is not only an intrinsic growth pathway in tumor cells but is also linked to epithelial differentiation, biosynthesis, stromal crosstalk, and immune tone. Normal prostate epithelium is maintained by luminal, basal, and specialized cell populations, and single-cell atlases of the adult prostate show that these states are more diverse than classical histology suggests (18, 47). In malignant tissue, AR activity is unevenly distributed across epithelial states. Some tumor cells show canonical luminal AR programs, whereas others display basal, club-like, hillock-like, inflammatory, stem-like, or neuroendocrine features (48, 49). This heterogeneity matters because ADT and ARSIs selectively inhibit AR-dependent cells while preserving or inducing states with low AR dependence, altered chromatin accessibility, or reliance on alternative survival signals.

In castration-resistant prostate cancer (CRPC), several resistance mechanisms still preserve AR addiction. AR amplification, mutation, enhancer remodeling, splice variants such as AR-V7, intratumoral androgen synthesis, and sustained AR target-gene expression can maintain growth under systemic androgen deprivation or AR blockade (50, 51). AR K609 acetylation provides an additional post-translational route to enzalutamide resistance and sustained AR signaling (52). Taxane outcomes are also influenced by prior ARSI exposure and treatment sequence: plasma AR status has been associated with docetaxel outcomes, and cabazitaxel outperformed a second ARSI after prior docetaxel and ARSI exposure (53, 54). Single-cell data further show that AR persistence is not uniform. The same tumor may contain AR-high cells sensitive to pathway inhibition, AR-low cells that persist, and intermediate-state cells that can re-enter AR programs under different pressures (3, 25). AR resistance is therefore not a binary switch but a distribution of cell states.

ADT can also reshape the immune ecosystem. Clinical and single-cell studies have shown that androgen deprivation can induce immune infiltration, antigen presentation changes, myeloid remodeling, and T-cell phenotype changes, which vary with disease stage and treatment background (17, 55). In metastatic castration-sensitive disease, combining anti-PD-1 therapy with ADT can induce substantial immune infiltration, yet durable clinical benefit remains limited to a subset of patients (17). In localized tumors, ADT can drive specific immune phenotypes, suggesting that endocrine therapy should be viewed as immune perturbation rather than just tumor cell perturbation (55). This observation supports rational sequencing of immunotherapy with androgen-axis treatment and suggests that immune activation may be accompanied by compensatory myeloid suppression.

AR signaling also coordinates metabolism. Androgen-regulated transcription can promote lipid synthesis, broader biosynthetic programs, and prostate-specific secretory functions (56, 57). In castration resistance, tumors can preserve or reshape these programs through SREBP-mediated cholesterol synthesis, *de novo* lipogenesis, CAMKK2-CREB signaling, or CHD1/SPOP-specific dependencies (14, 15). AR signaling can therefore organize a metabolic niche characterized by altered lipid handling and lactate-associated signaling (58, 59). These conditions may also shape immune function, although direct causal links to specific myeloid and T-cell states require further validation in prostate cancer. At the ecosystem level, resistance to AR-targeted therapy may reflect not only restoration of AR activity but also treatment-associated tissue changes that permit alternative metabolic and immune states to persist.

The dual effect of AR also explains why combination-therapy timing is difficult. Early ADT can reduce AR-dependent cells and transiently increase immune visibility, but the same pressure can select recurrent, lineage-plastic, or metabolically reprogrammed chromatin states (3, 17). More intensive AR blockade can prolong disease control while also favoring AR-low states that are less targetable by traditional endocrine strategies (60, 61). A rational ecosystem strategy should measure cell states during treatment rather than assume fixed baseline biology. If ADT induces T-cell infiltration without strong myeloid suppression, checkpoint-based combinations may be reasonable; if ADT enriches macrophage-fibroblast barriers or AR-low plastic cells, myeloid, stromal, epigenetic, or metabolic combinations may be more appropriate.

Earlier mechanistic reviews similarly concluded that CRPC often retains partial AR dependence through AR enhancer and binding-site remodeling, splice variants, co-regulator alterations, and non-canonical endocrine escape (62, 63). These mechanisms are best interpreted alongside single-cell state maps, as both AR-restorative and AR-independent resistant compartments can coexist within the same tumor.

### 3.2 Malignant epithelial cell states and lineage plasticity

Single-cell studies show that epithelial diversity in prostate cancer extends well beyond the traditional luminal-neuroendocrine dichotomy. Primary tumors contain multiple luminal subgroups, cribriform-associated states, pre-existing castration-resistant-like cells, club-like programs, and lineage-plastic populations (64, 65). Some states are low abundance, but clinically important because they can survive therapy, seed recurrence, or interact with immunosuppressive myeloid cells. Pre-existing castration-resistant-like cells in primary prostate cancer support a model in which ADT resistance can arise through selective expansion of a minority population rather than only through new evolution after treatment (3). Matched scATAC-seq/scRNA-seq analyses also identify pre-existing and persistent cells associated with relapse (24).

Lineage plasticity is a major resistance pathway. TP53 and RB1 deficiency, SOX2 activation, inflammatory JAK/STAT signaling, and epigenetic remodeling can weaken luminal AR-lineage constraints and promote treatment-emergent states (4, 66). ASCL1-dependent neuroendocrine differentiation and FOXA2-driven KIT pathway activation further define lineage-specific routes (5, 66). Neuroendocrine prostate cancer (NEPC) is not a single endpoint. Time-series and single-cell studies suggest branching trajectories, mixed states, and dynamic transitions through intermediate programs such as AR-low, stem-like, basal-like, or small-cell-like phenotypes (67, 68). These findings are clinically important because lineage-plastic tumors may lose PSMA, acquire DLL3 or other surface antigens, and alter DNA repair and replication programs. A recent multi-omics study developed an NEPC classifier and resolved molecularly distinct NEPC subtypes (26), whereas evidence for platinum-based chemotherapy combined with PD-1 blockade comes from a separate clinical study (69). It is important to note that cluster similarity, RNA velocity, and pseudotime analyses can suggest a plausible direction of transition but cannot, on their own, establish that one cell state actually gives rise to another; that inference requires longitudinal sampling of the same tumor, genetic lineage tracing, or matched epigenetic/clonal evidence. Among the studies cited above, direct longitudinal or lineage-tracing support comes mainly from model systems and small serial-biopsy cohorts [e.g., refs (24, 66)], whereas most human single-cell atlases capture only a cross-sectional snapshot of coexisting states.

Inflammatory context can actively promote plasticity. JAK/STAT signaling has been linked to prostate cancer lineage plasticity (4), and innate immunity and NF-κB pathways contribute to prostate stem-cell plasticity, reprogramming, and tumor initiation (70). These observations blur the boundary between tumor-intrinsic dedifferentiation and immune-microenvironmental pressure. Macrophage- or cytokine-rich niches may help generate or stabilize resistant epithelial states, although this remains a mechanistic hypothesis in many human datasets. Similarly, extracellular-matrix remodeling and fibroblast states can provide mechanical and growth-factor cues that support abnormal differentiation (19, 20).

Single-cell data also complicate biomarker development. A sample labeled “adenocarcinoma” by bulk biopsy may contain multiple epithelial states with different AR activity, metabolic dependence, and target-antigen expression. Therapy directed at PSMA, AR, PARP vulnerability, or neuroendocrine differentiation may therefore select neighboring states that lack the relevant target. This explains why spatial and single-cell target mapping are increasingly important for surface-antigen therapy, radioligand therapy, and lineage-directed assays (25, 71). The practical goal is not only to identify dominant clones, but also to detect resistant reserves: rare epithelial states positioned within stromal and immune neighborhoods that permit survival.

Resistant reserves are also a sampling problem. Standard biopsy can miss small but critical populations that are regionally restricted, treatment-induced, or enriched at invasion fronts and metastatic sites. A single-cell atlas of primary lesions is therefore necessary but insufficient to explain lethal prostate cancer. A patient may simultaneously harbor primary tumors, lymph-node lesions, and bone metastases, each with a distinct ecosystem. One lesion may remain AR-driven and PSMA-high, another may be AR-low and myeloid-rich, and a third may be neuroendocrine-like with different surface-antigen expression. Treatment chosen from one lesion may reduce target-positive disease while allowing hidden resistant states to expand. Spatially resolved multiregion sampling integrated with imaging and circulating biomarkers is a practical way to address this heterogeneity.

Epigenetic heterogeneity adds another layer to this sampling problem. DNA methylation, hydroxymethylation, noncoding RNA programs, and distinct chromatin states can generate phenotypic diversity that is difficult to detect by histology or single-site sequencing (72, 73). DNA hypomethylation-associated programs have also been reported to silence antitumor immune genes in early prostate cancer and circulating tumor cells, linking epigenetic state with immune visibility (74).

### 3.3 Metabolic reprogramming: lipids, hypoxia, and adaptive fuel use

Prostate cancer has a distinctive metabolic phenotype. Normal prostate epithelium accumulates citrate and has specialized secretory metabolism, whereas malignant transformation and progression shift cells toward lipid synthesis, cholesterol processing, oxidative metabolism, and biosynthetic growth (75, 76). AR signaling is closely linked to these programs and regulates enzymes and transcriptional networks that support lipogenesis and biosynthesis (46, 75). In CRPC, metabolic reprogramming can maintain growth even when androgen signaling is inhibited. Inhibition of *de novo* lipogenesis can target AR signaling in castration-resistant models (46), and recent studies implicate CAMKK2-CREB cholesterol metabolism, SREBP2-driven cholesterol synthesis after CHD1 loss, and hypoxia-induced lipid-droplet accumulation in ferroptosis resistance (14, 45).

Spatial omics adds an important dimension because metabolic dependence is not uniform within tumors. Spatial modeling of prostate cancer metabolic gene expression reveals extensive heterogeneity and candidate vulnerabilities (38). A review of spatial lipidomic and transcriptomic approaches highlights regional variation in lipid programs across tumor areas, stromal interfaces, and histological structures, but additional primary integrated datasets are needed (77). Hypoxia- and lactate-related biology, including HIF-1α-associated signaling and lactylation, may promote angiogenesis and vasculogenic mimicry (58). Studies in prostate cancer and other tumor systems suggest that lipid abundance, hypoxia, and lactate can influence macrophage and T-cell states; however, direct functional evidence linking these spatial metabolic features to defined immune-cell behavior in prostate cancer remains limited. Co-localization and pathway scores should therefore be viewed as hypothesis-generating unless supported by metabolite measurements, isotope tracing, or perturbation experiments.

Bone metastasis illustrates the ecosystem nature of metabolism. Bone is not only a destination for disseminated prostate cancer cells but also a metabolically active niche containing osteoblasts, osteoclasts, bone marrow immune cells, stromal cells, and mineralized matrix. Single-cell analysis of prostate cancer bone metastases has revealed an actionable immunosuppressive microenvironment (12), and studies of bone metastatic ecosystems show that myeloid-like tumor hybrid cells and bone-microenvironment signals can promote progression and re-dissemination (13). Spatial immune analysis of bone-metastatic CRPC also suggests that radiopharmaceutical therapy can modulate the bone-tumor microenvironment (29). These studies indicate that treatment response in bone metastasis may depend on local bone immune and metabolic conditions, not only on tumor genotype.

Profiling bone metastases carries specific technical constraints that can bias the cell-state estimates described above. Decalcification and bone-specific tissue processing can degrade RNA and protein, dissociation of mineralized tissue tends to under-recover tumor cells relative to marrow-resident populations, and needle or curettage sampling of a single small lesion region may not represent the whole metastatic deposit. Bone marrow contamination can also inflate apparent myeloid or hematopoietic content, and current bone-metastasis datasets are almost entirely dissociation-based, so the spatial architecture of the tumor-bone interface is usually lost; only a small number of studies [e.g., multiplex-imaging work in refs (28, 29)] preserve spatial information, and even these are limited to a few patients. Lesion-to-lesion heterogeneity within the same patient is well documented radiographically but rarely sampled molecularly, and paired primary-metastatic or metastasis-to-metastasis specimens remain scarce. Conclusions about the bone-adapted ecosystem should therefore be read as provisional until larger, spatially resolved, multi-lesion cohorts are available (**Table 1**).

Metabolic remodeling also intersects with resistance beyond AR therapy. Cancer-associated fibroblasts can regulate mitochondrial metabolism and reduce chemotherapy sensitivity through the ANGPTL4-IQGAP1 axis (44). Extracellular-matrix properties in organoid models can alter therapeutic responses to EZH2 and DRD2 inhibitors (78). Multi-omic integration has also identified mediators of olaparib resistance such as CRIP2 (79), while clinical PARP-inhibitor activity depends on DNA repair context (80). These examples show that metabolism, matrix, and DNA repair are not independent categories of resistance; they converge in cell-state adaptation. Tumor cells exposed to ARSIs, PARP inhibitors, radioligands, or chemotherapy must obtain fuel, manage oxidative stress, repair or tolerate damage, and evade immune clearance to survive.

Future metabolic research should move beyond pathway enrichment. Single-cell RNA sequencing can suggest fatty-acid synthesis, cholesterol metabolism, oxidative phosphorylation, hypoxia, or ferroptosis-resistance programs, but it cannot directly measure metabolite concentrations or flux. Spatial metabolomics, lipidomics, isotope tracing, high-throughput protein imaging, and organoid or explant perturbation are needed to determine whether inferred programs are true dependencies (38, 78). The strongest designs will connect spatial metabolic state, immune phenotype, and treatment outcome. For example, tumor regions rich in lipid droplets and resistant to ferroptosis should be evaluated for adjacency to immunosuppressive macrophages and for sensitivity to metabolic-immune combination therapy. Pathway- and gene-set-scoring approaches are also vulnerable to dropout, ambient RNA contamination, and gene-set composition effects, all of which can shift an inferred metabolic score independent of true biological signal; these technical sources of variation should be considered before a transcriptional metabolic program is treated as evidence of flux, substrate use, or dependency.

Metabolic interpretation should also consider systemic and host factors. Obesity, inflammation, and calcium and fatty-acid regulation are linked to prostate cancer biology (81, 82). These factors cannot substitute for tumor-cell metabolic analysis, but may help explain why genotypically similar tumors behave differently across patients.

The principal androgen-regulated epithelial and metabolic ecosystem states are summarized in **Table 2**.

**Table 2:** Summary of androgen-regulated epithelial and metabolic ecosystem states.

Ecosystem domain	Single-cell/spatial readouts	Resistance or therapeutic implication	Representative evidence
AR-dependent luminal programs	Heterogeneous AR-high and AR-low epithelial compartments in treatment-resistant disease.	Supports continued AR dependence in some cells while AR-low states persist under treatment pressure.	(3, 25)
Persistent and lineage-plastic epithelial states	Pre-existing castration-resistant-like cells and chromatin-defined persistent populations associated with relapse.	Creates a resistant reserve that may be missed by bulk biopsy and may require epigenetic, inflammatory, or lineage-directed combinations.	(3, 24)
Lipid and cholesterol metabolic rewiring	*De novo* lipogenesis, SREBP/cholesterol programs and context-specific metabolic dependencies across tumor regions.	Provides noncanonical survival routes after AR blockade and suggests metabolic co-targeting strategies.	(15, 46)
Hypoxia, lactate, and ferroptosis resistance	Spatially restricted hypoxia/lactylation signals, lipid droplet accumulation, and altered oxidative-stress tolerance.	Associated with angiogenesis and treatment-stress tolerance; direct immune effects require functional validation.	(45, 58)
Bone-adapted metabolic-immune niche	Tumor, myeloid, stromal, osteolineage, and marrow compartments arranged in metastatic bone ecosystems.	Therapy response may depend on local osteoimmune and radiopharmaceutical-responsive niches rather than tumor genotype alone.	(12, 29)

## 4 Immune, stromal, and spatial ecosystems

### 4.1 Immune and stromal architectures of prostate cancer

Prostate cancer is often described as an immune “cold” tumor, but single-cell and spatial studies show that this label is too coarse. Many tumors contain immune cells, but these cells are frequently organized into suppressive or ineffective states. Single-cell analysis of healthy and malignant prostate has resolved macrophages, T cells, B cells, mast cells, and stromal populations (16). Integrated single-cell and spatial transcriptomics have identified immunosuppressive myeloid populations and tissue neighborhoods that limit antitumor immunity (6). Other studies have described M2-associated macrophage markers (83), SPP1-positive macrophage and FAP-positive fibroblast crosstalk (20), CXCR4/CXCL12 signaling (84), and myeloid-mediated immunotherapy resistance (7, 27).

Macrophages are prominent components of immune suppression in prostate cancer. Single-cell studies resolve tumor-associated macrophages (TAMs) into distinct subpopulations, including SPP1-high macrophages with tumor-supportive features, FOLR2-positive tissue-resident macrophages, and perivascular macrophages with different cytokine programs (6, 28). SPP1-positive TAMs have been observed at tumor-stroma interfaces and in bone metastases, and multi-omic analyses suggest crosstalk with FAP-positive CAFs (20, 28). These spatial associations implicate macrophage-fibroblast neighborhoods in matrix remodeling, angiogenesis, and T-cell exclusion, but they do not establish a fixed set of soluble mediators in every context. Spatial transcriptomic studies further indicate that TAM density and state vary across invasion fronts, club-like epithelial regions, and abnormal vasculature (6, 10). A 2025 study reported that myeloid-mediated immunotherapy resistance evolves during treatment, with increasingly suppressive myeloid phenotypes under therapeutic pressure (27). This observation supports prospective testing of myeloid-targeted therapy before or together with checkpoint blockade. In preclinical prostate cancer models, IRE1α inhibition can reprogram TAMs toward an immunostimulatory phenotype (7), and the CXCR4/CXCL12 axis represents another candidate stromal-myeloid target (84).

T cells in prostate cancer occupy a spectrum of functional states, including cytotoxic effector, memory-like, exhausted, regulatory, and bystander phenotypes. Single-cell studies show that CD8+ T cells in prostate tumors frequently express inhibitory receptors such as PD-1, TIM-3, and LAG-3, defining an exhaustion-like phenotype associated with chronic antigen exposure and suppressive niche signals (6, 16). Receptor expression alone, however, does not establish functional exhaustion, which requires orthogonal functional validation. Spatial transcriptomics reveals that CD8+ T cells are often excluded from the tumor epithelial compartment and instead accumulate in peritumoral stroma, where CAF barriers and extracellular-matrix deposition limit direct cytotoxic contact (6). ADT can transiently increase immune infiltration by reducing androgen-mediated immune suppression and altering antigen-presentation programs (17, 55), but this benefit is context-dependent: anti-PD-1 therapy with ADT induced robust immune infiltration in metastatic castration-sensitive prostate cancer, yet durable benefit remains selective, suggesting that T-cell infiltration alone is insufficient without resolving myeloid and stromal suppression (17). A more useful immune classification should move beyond a hot/cold binary and distinguish immune-desert, myeloid-inflamed, T-cell-excluded, T-cell-exhausted, and treatment-remodeled states. Single-cell and spatial omics can assign tumors to these categories and identify which neighborhoods are permissive or restrictive for T-cell activity.

Other immune compartments remain less well characterized. Dendritic cells, natural killer cells, B cells, mast cells, and less abundant myeloid populations are detectable in prostate single-cell datasets (6, 16), whereas neutrophil- and myeloid-derived suppressor cell-like programs may be embedded within broader myeloid annotations and may change during therapy (27). Detection alone does not establish function: antigen presentation, cytotoxicity, antibody production, and suppressive activity require protein-level or perturbational validation. The macrophage- and T-cell-centered discussion in this review therefore reflects the depth of current evidence rather than the presumed absence of these other compartments.

**Figure 2:**
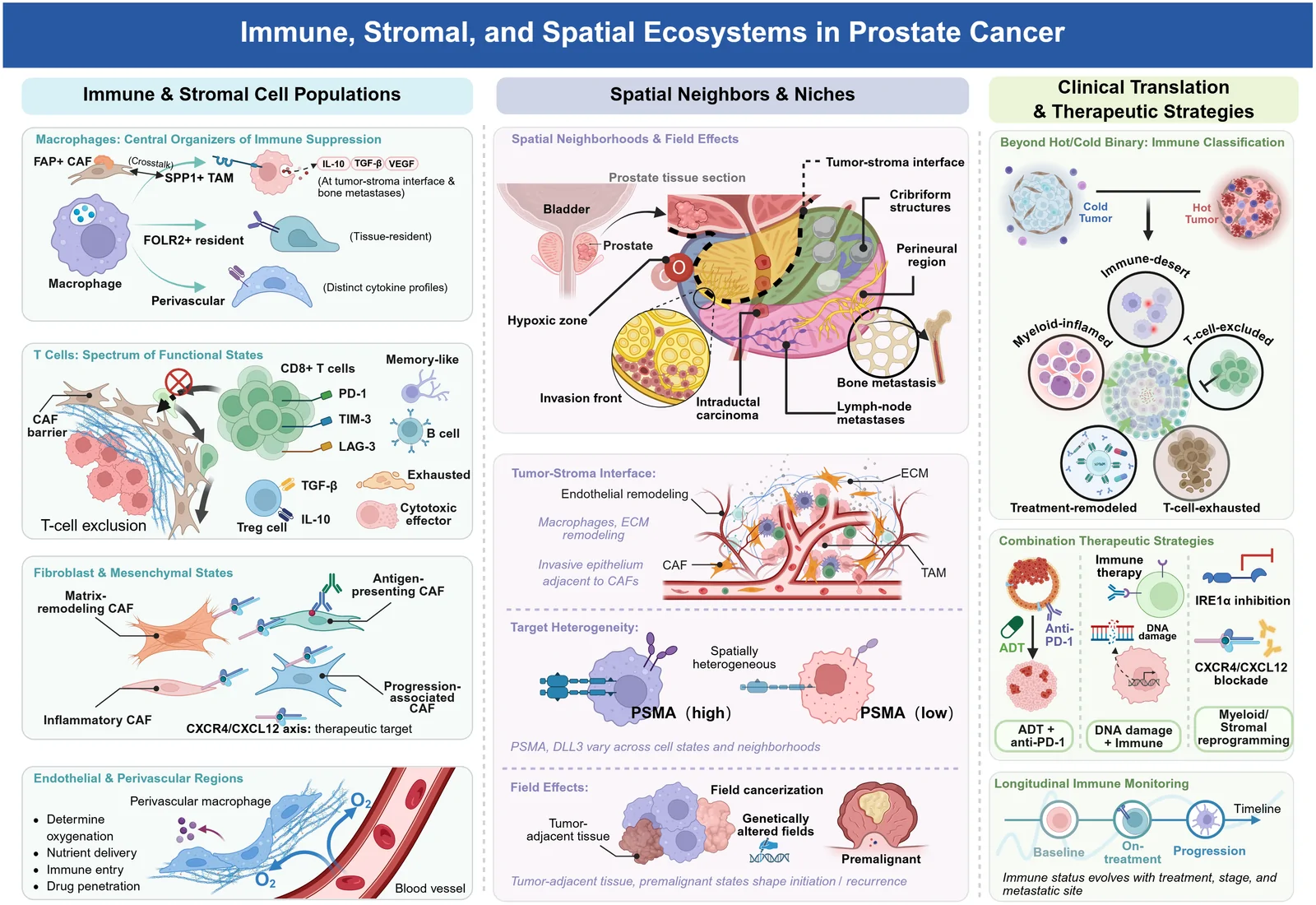
Immune, stromal, and spatial ecosystems in prostate cancer revealed by single-cell and spatial omics. The left panels summarize major macrophage, T-cell, fibroblast, endothelial, and perivascular states. The center panels illustrate representative spatial neighborhoods, tumor-stroma interfaces, therapeutic-target heterogeneity, and field effects. The right panels present a research framework for immune-state classification, mechanism-based combination therapy, and longitudinal monitoring. The depicted cellular interactions and spatial neighborhoods are context-dependent associations or computational predictions rather than validated clinical classifiers. The therapeutic strategies shown have different levels of evidence, ranging from clinically tested combinations, such as ADT plus anti-PD-1, to preclinical approaches, including IRE1α inhibition, CXCR4/CXCL12 blockade, and myeloid reprogramming. Their validation status is summarized in **Table 3** and discussed in the main text.

Fibroblast and mesenchymal states are equally important. Prostate CAFs can adopt matrix-remodeling, inflammatory, antigen-presentation-related, and progression-associated states (19, 30). CXCR4/CXCL12 crosstalk has been identified as a therapeutic axis in the prostate tumor microenvironment (84). SFRP4 and extracellular-matrix remodeling are spatially associated with prostate cancer (19). Distinct mesenchymal cell states can mediate disease progression (30), and multi-omics analyses have inferred signaling crosstalk between FAP-positive fibroblasts and SPP1-positive macrophages (20). These matrix programs are associated with immune exclusion, drug penetration, epithelial plasticity, and metabolic adaptation. In practice, a resistant neighborhood may contain AR-low epithelial cells, matrix-remodeling CAFs, inhibitory macrophages, abnormal vessels, hypoxia, and reduced antigen presentation. Single-cell and spatial omics make this composite phenotype visible (**Figure 2**).

Endothelial and perivascular regions also require more attention. Blood vessels determine oxygenation, nutrient delivery, immune entry, and drug exposure. Perivascular macrophages may shape local immune tone, while CXCR4/CXCL12 signaling links stromal and vascular remodeling to therapeutic vulnerability (84). Angiogenesis and vasculogenic-mimicry programs may be associated with hypoxia- and lactylation-related signals (58). In bone and visceral metastases, vascular structures also interact with organ-specific stromal cells. Tumors with similar genomic drivers may differ in treatment response depending on whether blood vessels permit drug penetration, whether perivascular niches recruit inhibitory macrophages, and whether hypoxic areas generate metabolic escape. Spatial omics is well suited to define these vascular-immune-metabolic relationships, but this area remains less developed than studies of epithelial and macrophage states.

Translating immune ecosystem findings into clinical decisions requires integration with treatment history. Immunotherapy for prostate cancer has historically shown limited activity in unselected populations, a pattern emphasized in prostate cancer immunotherapy reviews and tumor-microenvironment studies (85, 86). However, biomarker-enriched subsets and treatment-induced immune remodeling may create windows of vulnerability, underscoring the need for ecosystem-based stratification rather than histology-based selection alone (8, 17). Modern combination strategies increasingly target barriers identified by single-cell and spatial omics: pairing ADT with anti-PD-1 to leverage androgen-deprivation-induced immune infiltration (17), combining DNA damage response-targeted therapy with immune approaches where appropriate, and incorporating myeloid or stromal reprogramming to reduce TAM- or CAF-mediated T-cell suppression before checkpoint therapy (7, 84). The immune status of a prostate tumor is not static; it evolves with treatment, disease stage, and metastatic site, making longitudinal immune profiling essential for precision oncology. Where a candidate immune biomarker or spatial niche is discussed below, we indicate, where available, the cohort size, treatment context, assay platform, and whether the association has been validated in an independent or prospectively treated cohort; most current candidates remain single-cohort, hypothesis-generating associations rather than validated predictive biomarkers.

### 4.2 Spatial neighborhoods and field effects

Spatial analysis shifts attention from cell types to neighborhoods. A neighborhood is a recurrent spatial arrangement of epithelial, immune, stromal, vascular, and extracellular-matrix features that creates a local functional state. In prostate cancer, spatially resolved neighborhoods have been described in relation to intraductal carcinoma and age- or progression-associated tissue architectures (21, 22). Related work has highlighted benign field effects and field cancerization (87). Together, these studies suggest that tissue architecture and molecular state are closely linked and that histological patterns can serve as visible surrogates of underlying multicellular ecosystems.

An important spatial theme is the tumor-stroma interface. Invasive epithelial states often exist adjacent to CAFs, endothelial remodeling, macrophages, and extracellular-matrix programs rather than in isolation (6, 19). Matrix remodeling may provide structural support, growth factors, and immune exclusion. Vascular remodeling may alter oxygen delivery, drug accessibility, and immune trafficking. Hypoxia can induce lipid storage and ferroptosis resistance (45), while lactylation-associated programs may support angiogenesis (58). These local conditions may explain why genetically similar cells behave differently across regions of the same tumor.

Another theme is target heterogeneity. Surface antigens used for imaging or therapy, including PSMA and emerging lineage-related targets, can vary across cell states and neighborhoods (25, 88). Single-cell analysis of treatment-resistant prostate cancer emphasizes that cell-state changes can alter eligibility for surface-antigen-targeted therapy (25). Spatial analyses further suggest that radiopharmaceuticals may reshape the tumor microenvironment; however, the effects of PSMA-targeted radioligand therapy and bone-targeted radium-223 should be interpreted as distinct mechanisms (29, 71). Spatial target mapping should therefore be part of development pathways for radioligands, antibody-drug conjugates, bispecific antibodies, and cellular therapies.

Field effects may also have important implications. Spatial omics is beginning to reveal how tumor-adjacent tissue, genetically altered fields, inflammation, and benign epithelial states shape tumor initiation or recurrence (22, 87). ERG-driven tumor initiation is cell-context dependent (89), and normal prostate atlases show that different epithelial compartments have different regenerative and transformation potential (18, 47). Research on prevention and early intervention would therefore benefit from understanding not only malignant cells, but also the normal and premalignant tissue states from which they arise.

## 5 Therapy resistance and clinical translation

### 5.1 Therapy resistance across treatment modalities

Therapy resistance in prostate cancer is often discussed by drug category, but single-cell and spatial omics reveal common principles. First, resistance can pre-exist therapy: rare castration-resistant-like or persistent cells may be present before treatment and expand under selective pressure (3, 24). Second, resistance can be induced by treatment stress. Therapeutic pressure can reshape tumor and microenvironmental states, as shown in endocrine, immune, and radiopharmaceutical contexts (17, 71). Oncogenic evolution can also remodel neighboring epithelial, immune, and fibroblast states, as demonstrated in MYC-driven prostate carcinogenesis (90). Third, resistance is spatial. Clones occupying protective niches with favorable metabolism, matrix support, and immune escape may be more likely to survive. Critically, most available single-cell and spatial cohorts remain cross-sectional, comparing different patients before and after therapy rather than the same patient over time; only a minority of the studies discussed here include genuinely paired baseline/on-treatment sampling [e.g., refs (17, 29)], and this design distinction is indicated for each study in **Table 1**. Where a paired or longitudinal design is not available, differences between pretreatment and posttreatment groups are reported as associations rather than evidence of temporal evolution within a given tumor.

For ARSIs, resistance mechanisms include AR splice variants, AR amplification and mutation, co-regulator changes, intratumoral androgen synthesis, glucocorticoid or other nuclear-receptor compensation, lineage plasticity, and metabolic adaptation (39, 91). Single-cell studies further show that these mechanisms can coexist in different cells from the same patient (25). For taxanes, resistance may involve microtubule biology, AR transport, drug efflux, and cross-resistance after ARSI exposure (54, 92); CAF-mediated metabolic adaptation is supported by separate preclinical evidence (44). For PARP inhibitors, homologous recombination repair (HRR) alterations are not interchangeable: BRCA1/2 alterations show the most consistent sensitivity, whereas ATM and rarer alterations show less consistent responses (79, 93). Allelic status may further refine interpretation, but monoallelic versus biallelic inactivation has not been reported consistently across genes and trials. For PSMA-targeted radioligand therapy, antigen heterogeneity and variable target expression are established concerns, whereas altered DNA damage responses remain a proposed resistance mechanism that requires direct clinical validation (25, 94). Radium-223 is mechanistically distinct because it targets areas of increased bone turnover rather than PSMA; its reported immunomodulatory effects in bone metastases should not be generalized to PSMA-targeted therapy (29).

Pivotal trials provide the clinical context for these mechanisms. PROfound established olaparib after AR pathway inhibitor exposure in selected HRR-altered metastatic CRPC (93). In first-line metastatic CRPC, PROpel improved radiographic progression-free survival (rPFS), but its final prespecified overall survival (OS) analysis was not statistically significant (hazard ratio [HR] 0.81; p=0.054) (95). MAGNITUDE improved rPFS in the HRR-positive population, particularly in BRCA1/2-altered disease, and met a prespecified futility criterion in the HRR-negative cohort; however, its final analysis showed no significant OS difference in either the overall HRR-positive population or the BRCA1/2 subgroup (96). TALAPRO-2 improved rPFS and, at final analysis, significantly improved OS in the overall population (HR 0.80), with a stronger effect in HRR-deficient disease and no statistically significant OS difference in the HRR-non-deficient or unknown subgroup (97). Hematologic toxicity, particularly anemia, remains clinically relevant across PARP inhibitor combinations. For PSMA-targeted therapy, VISION demonstrated an OS benefit for 177Lu-PSMA-617 in previously treated PSMA-positive metastatic CRPC (98), and TheraP compared 177Lu-PSMA-617 with cabazitaxel in a randomized phase 2 setting (99). PSMAfore improved rPFS in taxane-naive metastatic CRPC after progression on one prior AR pathway inhibitor, but final intention-to-treat OS was not significantly different, with interpretation complicated by substantial crossover; crossover-adjusted analyses favored 177Lu-PSMA-617 (100). These trials support imaging- and clinicopathology-based selection but do not validate spatial-biopsy ecosystem classifiers for routine care.

Immunotherapy resistance follows a distinct ecosystem logic. Major barriers include low baseline immunogenicity in many prostate cancers, impaired antigen presentation, physical T-cell exclusion by CAFs and extracellular matrix, T-cell dysfunction driven by checkpoint ligands and suppressive cytokines, recruitment and polarization of immunosuppressive TAMs, regulatory T-cell (Treg) accumulation, and metabolic stress in lipid-rich or hypoxic regions that may weaken T-cell fitness, although direct prostate-specific functional evidence for the latter remains limited (6, 27). A 2025 Nature study showed that myeloid-mediated immunotherapy resistance is not a fixed baseline property but evolves during treatment, reframing resistance as a dynamic and partly systemic process rather than a purely local feature of the tumor microenvironment (27). Collectively, single-cell and spatial studies identify at least three poor-response configurations: immune-desert tumors with little T-cell entry, myeloid-inflamed tumors with high TAM density and physical T-cell exclusion, and T-cell-infiltrated tumors with exhausted effector programs (6, 27). Each configuration implies a different combination strategy. Immune-desert tumors may require stromal remodeling and innate immune activation; myeloid-inflamed tumors may require TAM reprogramming through pathways such as IRE1α or CXCR4/CXCL12 before checkpoint blockade; and exhausted T-cell tumors may require checkpoint combinations or metabolic interventions that restore T-cell function (7, 84). Prospective studies should evaluate whether on-treatment biopsy, spatial immune profiling, and circulating tumor DNA can distinguish these states and guide adaptive combinations more effectively than uniform checkpoint blockade.

Targeted therapy and immunotherapy also change how response should be assessed. Radiographic response may reduce dominant AR-dependent or PSMA-high populations while enriching smaller AR-low, PSMA-low, or neuroendocrine-like reserves. A sustained PSA decline can still mask expansion of plastic clones in PSA-low lineages. Conversely, immune infiltration on biopsy may be insufficient when effector cells remain spatially separated from tumor cells by inhibitory macrophage or CAF barriers. Response assessment should therefore integrate imaging, PSA kinetics, circulating tumor DNA, target-antigen mapping, and selective tissue reassessment. The clinical challenge is not only to determine whether treatment is working, but to identify the ecosystem that remains after treatment.

Neuroendocrine and aggressive-variant prostate cancers are especially challenging endpoints of resistance. These tumors can present with AR independence, TP53/RB1 loss, SOX2-related programs, epigenetic remodeling, and altered immune context (4, 66). FOXA2/KIT activation and ASCL1-linked programs further define lineage-specific states (5, 101). Platinum-based chemotherapy and checkpoint combinations may benefit selected patients, but durable control remains difficult (69). Single-cell and spatial analyses suggest that early detection of transitional states may be more valuable than treating fully established neuroendocrine disease. Capturing biomarkers of AR-low lineage plasticity, ASCL1/NEUROD1-like programs, DLL3 or KIT expression, and spatial myeloid suppression could guide earlier combination strategies (**Table 3**).

**Table 3:** Therapy resistance and clinical translation in prostate cancer metabolic-immune ecosystems.

Therapeutic pressure	Dominant resistance ecosystem	Single-cell/spatial omics readouts	Clinical translation	Refs.
ADT and ARSIs	Pre-existing persistent cells and lineage-plastic escape under androgen-axis pressure.	AR-high and AR-low compartments; persistent chromatin states; epithelial plasticity programs; spatially protected resistant reserves.	Evaluate on-treatment profiling prospectively to distinguish continued AR dependence from AR-low plastic escape before sequencing additional AR-targeted therapy.	(3, 24)
Taxanes	Treatment-sequence-related cross-resistance, drug efflux, and proposed microtubule/AR-trafficking changes.	Residual proliferative and AR-active tumor states interpreted in the context of prior ARSI and taxane exposure.	Interpret taxane response alongside treatment sequence and tumor cell-state markers rather than relying only on PSA decline.	(54, 92)
PARP inhibitors	HRR alterations are not interchangeable; BRCA1/2 status and acquired or secondary resistance can influence response.	Gene-specific HRR status, DNA repair-defective states, and molecular evidence of acquired resistance.	Use validated gene-specific biomarkers; ecosystem markers remain investigational for patient selection.	(79, 93)
PSMA radioligand therapy	PSMA heterogeneity, antigen-low reserves, and variable target expression.	Intrapatient PSMA distribution; PSMA-low or neuroendocrine-like cells; imaging-defined target heterogeneity.	Use PSMA imaging and treatment history for eligibility; spatial ecosystem classifiers remain investigational.	(25, 98)
Bone-targeted alpha therapy (radium-223)	Bone-dominant disease and treatment-associated remodeling of the bone tumor microenvironment.	Spatial immune changes in bone metastases after radium-223 exposure.	Interpret radium-223-associated immune remodeling separately from PSMA-targeted radioligand resistance.	(29)
Immune checkpoint blockade	T-cell exclusion or exhaustion-like states and suppressive macrophage programs.	Myeloid-inflamed, T-cell-excluded, or T-cell-exhausted spatial neighborhoods.	Evaluate immune-active subsets prospectively and test checkpoint blockade with mechanism-matched myeloid strategies.	(6, 27)
Platinum and neuroendocrine-directed therapy	AR independence, bifurcating neuroendocrine lineage programs, FOXA2/KIT activation, and epigenetic remodeling.	Transition states between adenocarcinoma and neuroendocrine-like phenotypes; lineage markers and antigen shifts.	Detect AR-low transitions earlier and test lineage-directed combinations before fully established neuroendocrine disease.	(5, 66)
Prospective adaptive research framework	Proposed framework: treatment may leave target-negative or niche-protected populations rather than a single resistant clone.	Prospective baseline, early on-treatment, and progression sampling integrated with circulating tumor DNA (ctDNA), circulating tumor cells (CTCs), imaging, and spatial target maps.	Test whether mechanism-matched tissue endpoints can show that the intended resistant ecosystem was altered.	(25, 88)

### 5.2 Translational opportunities

The translational value of single-cell and spatial omics lies in converting complex atlases into candidate classifiers. Candidate assays may quantify resistant epithelial states and lineage programs (25, 26), myeloid-associated resistance or predictive signatures (27, 102), CAF-associated matrix programs (19, 20), and spatial metabolic vulnerabilities (38). Spatial information is especially important when therapy requires target homogeneity or immune accessibility. For example, research should evaluate whether spatial measures of PSMA heterogeneity add predictive value beyond validated PSMA imaging; spatial-biopsy classifiers are not yet established for excluding patients from radioligand therapy (25, 71).

Combination hypotheses should be matched to their evidence level. Human single-cell and spatial cohorts primarily support associations, organoid and mouse models provide mechanistic tests, and only a subset of strategies has entered clinical trials. Tumors with high lipid- or cholesterol-synthesis programs provide a rationale for testing metabolic combinations with AR blockade (14, 46). AR-low plastic states motivate evaluation of inflammatory or epigenetic strategies (4) and of lineage-directed approaches involving KIT or ASCL1 (5, 101). Myeloid-rich or CAF-rich tumors motivate testing macrophage, CXCR4/CXCL12, or stroma-directed interventions (7, 84). Bone metastases may require approaches that separately address tumor cells and osteoimmune niches (12, 13), while distinguishing the effects of bone-targeted radium-223 from PSMA-targeted radioligand therapy (29, 98). None of these ecosystem classifiers or mechanism-matched combinations is yet prospectively validated for routine treatment selection.

Longitudinal sampling is crucial. A single baseline biopsy cannot capture the adaptive pathways that emerge after ADT, ARSIs, PARP inhibitors, taxanes, radioligand therapy, or immunotherapy. When feasible, future clinical trials should prospectively collect pretreatment, early on-treatment, and progression samples and integrate tissue findings with circulating tumor DNA, circulating tumor cells, extracellular vesicles, imaging, and clinical outcomes. This proposed design would help distinguish expected target loss, niche protection of target-positive cells, expansion of rare target-negative states, and treatment-induced suppressive ecosystems.

Clinical implementation must also be realistic. Whole-transcriptome spatial profiling and single-cell sequencing are unlikely to be routine for every patient in the near term. High-dimensional omics can support mechanism discovery, after which compact tissue assays such as multiplex immunohistochemistry, targeted spatial RNA panels, and digital pathology classifiers can be developed and validated. Blood- and urine-based biomarkers offer complementary, less invasive options (103). The best assays should measure mechanisms, not only descriptive cell labels. A research report might summarize AR-high luminal disease with a small AR-low basal-like/hillock-like reserve, PSMA heterogeneity, an SPP1-positive macrophage/FAP-positive fibroblast niche, a high cholesterol-synthesis signature, and low antigen presentation. Whether such a format improves treatment selection over conventional reporting requires prospective validation.

Experimental design should match this biology. Rather than testing a combination regimen in all metastatic CRPC, future trials could prospectively evaluate enrichment for defined ecosystems: tumors with AR-low plastic cells and JAK/STAT activity after ARSI therapy; PSMA-heterogeneous bone metastases; myeloid-inflamed tumors with SPP1-positive macrophages and FAP-positive fibroblast neighborhoods; or CRPC with lipid-synthesis programs and immune exclusion. Endpoints should include not only PSA response and imaging progression-free survival, but also whether the intended ecosystem was altered. If macrophage-targeted therapy fails to reduce the inhibitory macrophage neighborhood, a negative trial would not by itself refute the underlying myeloid hypothesis because target engagement was not demonstrated. Mechanism-matched tissue endpoints are essential for learning from both success and failure.

### 5.3 Limitations and future directions

Despite rapid progress, the field should avoid overinterpreting cell atlases. Cell clusters are not necessarily stable biological entities; they may reflect sampling, dissociation, batch effects, treatment timing, or computational choices. Pathway scores are approximations, especially for metabolism, where mRNA abundance does not equal flux. Spatial transcriptomics may infer cell types in mixed spots, whereas single-cell sequencing may lose spatially anchored populations. Many studies have limited sample sizes, incomplete coverage of heavily treated metastatic disease, and insufficient clinical follow-up. The next stage requires standardized tissue processing, pathological annotation, multiregion sampling, matched germline and somatic genomics, spatial validation, and shared data models.

The integration of metabolism and immunity remains insufficient. Transcriptional signatures of lipid synthesis, hypoxia, lactate signaling, oxidative phosphorylation, or ferroptosis resistance need to be linked with metabolomics, isotope tracing, imaging mass spectrometry, and functional perturbation. Similarly, inferred ligand-receptor interactions among tumor cells, CAFs, macrophages, and T cells require orthogonal functional validation. Organoids, matrix-informed tissue models, and CAF-focused co-culture systems provide practical platforms for this purpose (78, 104). Computational models should integrate spatial distance, pathway activity, clonal identity, antigen expression, and therapeutic exposure rather than treating each modality separately. Different ligand-receptor or cell-communication algorithms can assign discordant interaction partners and directionality to the same dataset; computationally inferred communication networks discussed in this review (**Table 1**) should therefore be reported alongside the method used and treated as hypotheses pending protein-level, co-culture, or perturbation-based validation.

Data sharing and nomenclature also need standardization. Different studies may use different names for similar luminal, basal, club-like, hillock-like, macrophage, or CAF states. Without a shared reference, the same biological state may appear as multiple unrelated clusters, while different treatment-induced states may be grouped under familiar labels. Community reference maps should include normal prostate, untreated localized cancer, tumors after neoadjuvant therapy, metastatic castration-sensitive disease, CRPC, neuroendocrine disease, and organ-specific metastases. These maps should be paired with minimum biomarker panels that can be implemented in pathology laboratories. The goal is not to force every tumor into one category, but to create a common language for cross-cohort and trial comparison.

Future research should prioritize three questions. First, what is the earliest detectable resistant state after androgen-pathway inhibition, and can it be cleared before clinical progression? Second, which spatial neighborhoods predict response or resistance to immunotherapy, PARP inhibitors, taxanes, and PSMA radioligand therapy? Third, can metabolic interventions reprogram the immune niche rather than merely inhibit tumor growth? Answering these questions will require coordinated longitudinal cohorts, clinically annotated serial biopsies, and perturbation-based validation.

### 5.4 A practical working model

As a proposed descriptive framework rather than a validated clinical taxonomy, the metabolic-immune ecosystem in prostate cancer can be organized around four interconnected levels: malignant epithelial state, metabolic program, immune-stromal neighborhood, and treatment pressure. At the epithelial level, dominant and minority tumor populations should be characterized according to luminal AR-high, AR-low persistent, basal-like, club-like, hillock-like, lineage-plastic, neuroendocrine-like, and mixed transitional programs (24, 105). At the metabolic level, investigators should ask whether these populations rely on lipid synthesis, cholesterol synthesis, oxidative phosphorylation, hypoxia adaptation, ferroptosis resistance, or bone-niche resources (38, 45). At the immune-stromal level, the key question is whether the tumor exhibits an immune-desert, T-cell-infiltrated, macrophage-dominant, CAF-rich/T-cell-excluded, antigen-presentation-deficient, perivascularly suppressed, or bone-niche-specialized ecosystem (6, 12). At the treatment-pressure level, the question is which ecosystem has been shaped by prior therapies and which resistant reserves remain (24, 25).

Several recurring ecosystem states can be proposed from this framework. The first is an AR-high metabolic luminal ecosystem, in which tumors retain luminal differentiation, AR target-gene expression, and androgen-regulated biosynthesis. These tumors may respond to ADT and ARSIs but recur through AR restoration or splice variants (50, 51). Cholesterol reprogramming and persistent cell states may provide additional routes to resistance (15, 24). The second is an AR-low plastic ecosystem, characterized by reduced dependence on classical AR signaling and enrichment of stem-like, basal-like, club-like, inflammatory, or neuroendocrine-related features (4, 105). Persistent AR addiction and AR variation remain important escape routes that should still be monitored (106, 107). The third is a myeloid-stromal suppressive ecosystem in which macrophages, CAFs, extracellular matrix, CXCL12/CXCR4 signaling, SPP1-FAP interactions, hypoxia, and abnormal vasculature are associated with restricted immune access and potentially reduced drug penetration (20, 84). The fourth is a bone-adapted ecosystem in which tumor cells interact with bone immune cells, bone marrow stroma, and radiopharmaceutical-responsive niches (12, 29). These states are not mutually exclusive; different lesions in the same patient may carry different ecosystems.

This model also explains why monotherapy often produces partial and temporary control. ARSIs can inhibit AR-high ecosystems while leaving AR-low plastic and myeloid-protected reserves. PARP inhibitors can benefit selected DNA repair-defective tumors, but resistance may emerge through secondary mediators such as CRIP2 (79, 80). PSMA radioligand therapy can kill PSMA-high regions while sparing antigen-low or neuroendocrine-like cells (25, 71). Checkpoint blockade may fail when T cells are absent, excluded, exhausted, or metabolically impaired (17, 27). Chemotherapy can reduce proliferative burden, while CAF-mediated mitochondrial adaptation can reduce drug sensitivity (44); treatment history and sequencing remain clinically important (2). Treatment should therefore be viewed as ecosystem editing: the key issue is not only which cells are killed, but which niches and cell states are left to repopulate the disease.

In research practice, this working model suggests that high-dimensional studies should report at least disease state, treatment exposure, lesion site, pathological pattern, genomic driver, AR-pathway status, dominant and minority epithelial states, immune-stromal composition, spatial neighborhood, and outcome. Each resistant state should be tested for target expression and metabolic vulnerability rather than merely named. Whenever feasible, baseline and progression samples from the same patient should be compared. This is particularly important for neuroendocrine transformation and myeloid-mediated immunotherapy resistance, because cross-sectional comparisons can confuse pre-existing biology with treatment-induced evolution (27, 66). Functional validation should prioritize translational perturbations such as AR-pathway modulation, lipid or cholesterol targeting, macrophage reprogramming, CXCR4/CXCL12 blockade, epigenetic therapy, PARP inhibition, radioligand therapy, and lineage-directed surface-antigen targeting.

In clinical practice, this model points toward future ecosystem reporting. Such reports should not replace histology, PSA, imaging, germline testing, somatic tumor sequencing, or treatment guidelines; instead, they should provide context for difficult decisions: whether to use another ARSI after progression, whether to prioritize a taxane or PARP inhibitor, whether PSMA radioligand therapy may be weakened by antigen heterogeneity, whether immunotherapy trials should include myeloid or stromal targeting, and whether the patient is developing an AR-low neuroendocrine trajectory. These reports could be generated from targeted spatial panels, multiplex protein imaging, digital pathology, blood biomarkers, and selected single-cell datasets. Their value requires prospective validation, but the biological rationale is strong: prostate cancer progression is not isolated clonal evolution, but adaptation within a changing metabolic-immune tissue system.

The model also emphasizes the difference between target discovery and resistance ecology. Target discovery asks whether a molecule or pathway can be inhibited; resistance ecology asks whether the inhibited population determines the patient’s next progression event. A target may be biologically plausible but spatially restricted, absent from rare resistant states, or embedded in a niche that restores growth through compensatory signals. Conversely, a moderately expressed target may be clinically valuable if it is stable in small populations that repeatedly disseminate and recur. The strength of single-cell and spatial omics lies in detecting these mismatches before therapies are widely tested. Candidate targets should therefore be evaluated by three criteria: whether they are expressed in clinically dangerous states, whether they are spatially accessible in resistant neighborhoods, and whether they fit rational combination therapy that also alters the surrounding metabolic-immune context.

## 6 Conclusion

Single-cell and spatial omics have moved prostate cancer biology beyond linear AR-resistance models. AR signaling remains central, but it operates within a multicellular ecosystem shaped by epithelial plasticity, lipid and cholesterol metabolism, hypoxia, fibroblast remodeling, macrophage suppression, T-cell dysfunction, bone-niche biology, and treatment selection pressure. A clinically important hypothesis is that resistance may reside in rare cell states or protected neighborhoods before it becomes visible as radiographic progression. Future precision treatment may benefit from detecting these therapy-resistant ecosystems early and matching combination therapy to their vulnerabilities. The field already has tools to map these ecosystems; the next challenge is to turn maps into decisions.

In practice, the most valuable studies will connect spatially localized resistant cell states with metabolic dependence, immune escape mechanisms, and treatment decisions. This standard requires moving beyond descriptive atlas construction toward prospective validation before ecosystem biology can improve patient care.

## References

[1] Swami U McFarland TR Nussenzveig R Agarwal N . Advanced prostate cancer: Treatment advances and future directions. Trends Cancer. (2020) 6:702–15. doi: 10.1016/j.trecan.2020.04.010 32534790

[2] de Bono JS Oudard S Ozguroglu M Hansen S Machiels JP Kocak I . Prednisone plus cabazitaxel or mitoxantrone for metastatic castration-resistant prostate cancer progressing after docetaxel treatment: a randomised open-label trial. Lancet. (2010) 376:1147–54. doi: 10.1016/S0140-6736(10)61389-X 20888992

[3] Cheng Q Butler W Zhou Y Zhang H Tang L Perkinson K . Pre-existing castration-resistant prostate cancer-like cells in primary prostate cancer promote resistance to hormonal therapy. Eur Urol. (2022) 81:446–55. doi: 10.1016/j.eururo.2021.12.039 35058087 PMC9018600

[4] Chan JM Zaidi S Love JR Zhao JL Setty M Wadosky KM . Lineage plasticity in prostate cancer depends on JAK/STAT inflammatory signaling. Science. (2022) 377:1180–91. doi: 10.1126/science.abn0478 35981096 PMC9653178

[5] Han M Li F Zhang Y Dai P He J Li Y . FOXA2 drives lineage plasticity and KIT pathway activation in neuroendocrine prostate cancer. Cancer Cell. (2022) 40:1306–23.e8. doi: 10.1016/j.ccell.2022.10.011 36332622

[6] Hirz T Mei S Sarkar H Kfoury Y Wu S Verhoeven BM . Dissecting the immune suppressive human prostate tumor microenvironment via integrated single-cell and spatial transcriptomic analyses. Nat Commun. (2023) 14:663. doi: 10.1038/s41467-023-36325-2 36750562 PMC9905093

[7] Unal B Kuzu OF Jin Y Osorio D Kildal W Pradhan M . Targeting IRE1alpha reprograms the tumor microenvironment and enhances anti-tumor immunity in prostate cancer. Nat Commun. (2024) 15:8895. doi: 10.1038/s41467-024-53039-1 39406723 PMC11480464

[8] Abida W Cyrta J Heller G Prandi D Armenia J Coleman I . Genomic correlates of clinical outcome in advanced prostate cancer. Proc Natl Acad Sci USA. (2019) 116:11428–36. doi: 10.1073/pnas.1902651116 31061129 PMC6561293

[9] Adams EJ Karthaus WR Hoover E Liu D Gruet A Zhang Z . FOXA1 mutations alter pioneering activity, differentiation and prostate cancer phenotypes. Nature. (2019) 571:408–12. doi: 10.1038/s41586-019-1318-9 31243370 PMC6661172

[10] Kiviaho A Eerola SK Kallio HML Andersen MK Hoikka M Tiihonen AM . Single cell and spatial transcriptomics highlight the interaction of club-like cells with immunosuppressive myeloid cells in prostate cancer. Nat Commun. (2024) 15:9949. doi: 10.1038/s41467-024-54364-1 39550375 PMC11569175

[11] Bian X Wang W Abudurexiti M Zhang X Ma W Shi G . Integration analysis of single-cell multi-omics reveals prostate cancer heterogeneity. Adv Sci (Weinh). (2024) 11:e2305724. doi: 10.1002/advs.202305724 38483933 PMC11095148

[12] Kfoury Y Baryawno N Severe N Mei S Gustafsson K Hirz T . Human prostate cancer bone metastases have an actionable immunosuppressive microenvironment. Cancer Cell. (2021) 39:1464–78.e8. doi: 10.1016/j.ccell.2021.09.005 34719426 PMC8578470

[13] Ye X Huang X Fu X Zhang X Lin R Zhang W . Myeloid-like tumor hybrid cells in bone marrow promote progression of prostate cancer bone metastasis. J Hematol Oncol. (2023) 16:46. doi: 10.1186/s13045-023-01442-4 37138326 PMC10155318

[14] Lin C Pulliam TL Han JJ Xu J Recio CV Wilkenfeld SR . Cholesterol metabolism regulated by CAMKK2-CREB signaling promotes castration-resistant prostate cancer. Cell Rep. (2025) 44:115792. doi: 10.1016/j.celrep.2025.115792 40483692 PMC12289408

[15] Chen F Li H Wang Y Tang X Lin K Li Q . CHD1 loss reprograms SREBP2-driven cholesterol synthesis to fuel androgen-responsive growth and castration resistance in SPOP-mutated prostate tumors. Nat Cancer. (2025) 6:854–73. doi: 10.1038/s43018-025-00952-z 40360905 PMC12276840

[16] Tuong ZK Loudon KW Berry B Richoz N Jones J Tan X . Resolving the immune landscape of human prostate at a single-cell level in health and cancer. Cell Rep. (2021) 37:110132. doi: 10.1016/j.celrep.2021.110132 34936871 PMC8721283

[17] Hawley JE Obradovic AZ Dallos MC Lim EA Runcie K Ager CR . Anti-PD-1 immunotherapy with androgen deprivation therapy induces robust immune infiltration in metastatic castration-sensitive prostate cancer. Cancer Cell. (2023) 41:1972–88.e5. doi: 10.1016/j.ccell.2023.10.006 37922910 PMC11184948

[18] Karthaus WR Hofree M Choi D Linton EL Turkekul M Bejnood A . Regenerative potential of prostate luminal cells revealed by single-cell analysis. Science. (2020) 368:497–505. doi: 10.1126/science.aay0267 32355025 PMC7313621

[19] Andersen MK Krossa S Midtbust E Pedersen CA Wess M Høiem TS . Spatial transcriptomics reveals strong association between SFRP4 and extracellular matrix remodeling in prostate cancer. Commun Biol. (2024) 7:1462. doi: 10.1038/s42003-024-07161-x 39511287 PMC11543834

[20] Wu T Li X Zheng F Liu H Yu Y . Intercellular communication between FAP+ fibroblasts and SPP1+ macrophages in prostate cancer via multi-omics. Front Immunol. (2025) 16:1560998. doi: 10.3389/fimmu.2025.1560998 40438108 PMC12116517

[21] Takahara T Taniguchi N Sassa N Tsuzuki T . Spatial transcriptomics of intraductal carcinoma of the prostate. Histopathology. (2025) 87:745–56. doi: 10.1111/his.15551 40964799

[22] Cheng Y Liu B Xin J Wu X Li W Shang J . Single-cell and spatial RNA sequencing identify divergent microenvironments and progression signatures in early- versus late-onset prostate cancer. Nat Aging. (2025) 5:909–28. doi: 10.1038/s43587-025-00842-0 40211000

[23] Cheng Y Liu B Xin J Wu X Li W Shang J . Author correction: Single-cell and spatial RNA sequencing identify divergent microenvironments and progression signatures in early- versus late-onset prostate cancer. Nat Aging. (2025) 5:1370–1. doi: 10.1038/s43587-025-00892-4 40355759

[24] Taavitsainen S Engedal N Cao S Handle F Erickson A Prekovic S . Single-cell ATAC and RNA sequencing reveal pre-existing and persistent cells associated with prostate cancer relapse. Nat Commun. (2021) 12:5307. doi: 10.1038/s41467-021-25624-1 34489465 PMC8421417

[25] Zaidi S Park J Chan JM Roudier MP Zhao JL Gopalan A . Single-cell analysis of treatment-resistant prostate cancer: Implications of cell state changes for cell surface antigen-targeted therapies. Proc Natl Acad Sci USA. (2024) 121:e2322203121. doi: 10.1073/pnas.2322203121 38968122 PMC11252802

[26] Shen J Lu L Chen Z Guo W Wang S Liu Z . Multi-omics analysis constructs a novel neuroendocrine prostate cancer classifier and classification system. Sci Rep. (2025) 15:13901. doi: 10.1038/s41598-025-96683-3 40263498 PMC12015331

[27] Lyu A Fan Z Clark M Lea A Luong D Setayesh A . Evolution of myeloid-mediated immunotherapy resistance in prostate cancer. Nature. (2025) 637:1207–17. doi: 10.1038/s41586-024-08290-3 39633050 PMC11779626

[28] Mei S Zhang H Hirz T Jeffries NE Xu Y Baryawno N . Single-cell and spatial transcriptomics reveal a tumor-associated macrophage subpopulation that mediates prostate cancer progression and metastasis. Mol Cancer Res. (2025) 23:653–65. doi: 10.1158/1541-7786.MCR-24-0791 40105746 PMC12221797

[29] Corn PG Yu G Fan Y Zhang M Troncoso P Surasi DS . Spatial immune profiling of bone-metastatic castration-resistant prostate cancer reveals radium-223 immunomodulates the bone-tumor microenvironment. Prostate Cancer Prostatic Dis. (2025). doi: 10.1038/s41391-025-01069-1 41454189 PMC13472971

[30] Pakula H Omar M Carelli R Pederzoli F Fanelli GN Pannellini T . Distinct mesenchymal cell states mediate prostate cancer progression. Nat Commun. (2024) 15:363. doi: 10.1038/s41467-023-44210-1 38191471 PMC10774315

[31] Xin S Liu X Li Z Sun X Wang R Zhang Z . ScRNA-seq revealed an immunosuppression state and tumor microenvironment heterogeneity related to lymph node metastasis in prostate cancer. Exp Hematol Oncol. (2023) 12:49. doi: 10.1186/s40164-023-00407-0 37221625 PMC10204220

[32] Xin S Liu X Li Z Sun X Wang R Zhang Z . Correction: ScRNA-seq revealed an immunosuppression state and tumor microenvironment heterogeneity related to lymph node metastasis in prostate cancer. Exp Hematol Oncol. (2024) 13:54. doi: 10.1186/s40164-024-00517-3 38769580 PMC11103884

[33] Macosko EZ Basu A Satija R Nemesh J Shekhar K Goldman M . Highly parallel genome-wide expression profiling of individual cells using nanoliter droplets. Cell. (2015) 161:1202–14. doi: 10.1016/j.cell.2015.05.002 26000488 PMC4481139

[34] Satija R Farrell JA Gennert D Schier AF Regev A . Spatial reconstruction of single-cell gene expression data. Nat Biotechnol. (2015) 33:495–502. doi: 10.1038/nbt.3192 25867923 PMC4430369

[35] Tabula Muris Consortium . Single-cell transcriptomics of 20 mouse organs creates a Tabula Muris. Nature. (2018) 562:367–72. doi: 10.1038/s41586-018-0590-4 30283141 PMC6642641

[36] Dong B Miao J Wang Y Luo W Ji Z Lai H . Single-cell analysis supports a luminal-neuroendocrine transdifferentiation in human prostate cancer. Commun Biol. (2020) 3:778. doi: 10.1038/s42003-020-01476-1 33328604 PMC7745034

[37] Ståhl PL Salmén F Vickovic S Lundmark A Navarro JF Magnusson J . Visualization and analysis of gene expression in tissue sections by spatial transcriptomics. Science. (2016) 353:78–82. doi: 10.1126/science.aaf2403 27365449

[38] Wang Y Ma S Ruzzo WL . Spatial modeling of prostate cancer metabolic gene expression reveals extensive heterogeneity and selective vulnerabilities. Sci Rep. (2020) 10:3490. doi: 10.1038/s41598-020-60384-w 32103057 PMC7044328

[39] Antonarakis ES Lu C Wang H Luber B Nakazawa M Roeser JC . AR-V7 and resistance to enzalutamide and abiraterone in prostate cancer. N Engl J Med. (2014) 371:1028–38. doi: 10.1056/NEJMoa1315815 25184630 PMC4201502

[40] Nakazawa M Antonarakis ES Luo J . Androgen receptor splice variants in the era of enzalutamide and abiraterone. Horm Cancer. (2014) 5:265–73. doi: 10.1007/s12672-014-0190-1 25048254 PMC4167475

[41] Feng DC Zhu WZ Wang J Li DX Shi X Xiong Q . The implications of single-cell RNA-seq analysis in prostate cancer: Unraveling tumor heterogeneity, therapeutic implications and pathways towards personalized therapy. Mil Med Res. (2024) 11:21. doi: 10.1186/s40779-024-00526-7 38605399 PMC11007901

[42] Granja JM Corces MR Pierce SE Bagdatli ST Choudhry H Chang HY . ArchR is a scalable software package for integrative single-cell chromatin accessibility analysis. Nat Genet. (2021) 53:403–411. doi: 10.1038/s41588-021-00790-6 33633365 PMC8012210

[43] Giesen C Wang HA Schapiro D Zivanovic N Jacobs A Hattendorf B . Highly multiplexed imaging of tumor tissues with subcellular resolution by mass cytometry. Nat Methods. (2014) 11:417–22. doi: 10.1038/nmeth.2869 24584193

[44] Xiong Z Zhuang RL Yu SL Xie ZX Peng SR Li ZA . Cancer-associated fibroblasts regulate mitochondrial metabolism and inhibit chemosensitivity via ANGPTL4-IQGAP1 axis in prostate cancer. J Adv Res. (2025) 75:663–78. doi: 10.1016/j.jare.2024.12.003 39647634 PMC12789717

[45] Chauhan SS Vizzerra AD Liou H Flores CE Snider AJ Snider JM . Hypoxia induced lipid droplet accumulation promotes resistance to ferroptosis in prostate cancer. Oncotarget. (2025) 16:532–44. doi: 10.18632/oncotarget.28750 40560062 PMC12196323

[46] Zadra G Ribeiro CF Chetta P Ho Y Cacciatore S Gao X . Inhibition of de novo lipogenesis targets androgen receptor signaling in castration-resistant prostate cancer. Proc Natl Acad Sci USA. (2019) 116:631–40. doi: 10.1073/pnas.1808834116 30578319 PMC6329966

[47] Henry GH Malewska A Joseph DB Malladi VS Lee J Torrealba J . A cellular anatomy of the normal adult human prostate and prostatic urethra. Cell Rep. (2018) 25:3530–42.e5. doi: 10.1016/j.celrep.2018.11.086 30566875 PMC6411034

[48] Chen S Zhu G Yang Y Wang F Xiao YT Zhang N . Single-cell analysis reveals transcriptomic remodellings in distinct cell types that contribute to human prostate cancer progression. Nat Cell Biol. (2021) 23:87–98. doi: 10.1038/s41556-020-00613-6 33420488

[49] Song H Weinstein HNW Allegakoen P Wadsworth MH Xie J Yang H . Single-cell analysis of human primary prostate cancer reveals the heterogeneity of tumor-associated epithelial cell states. Nat Commun. (2022) 13:141. doi: 10.1038/s41467-021-27322-4 35013146 PMC8748675

[50] Sprenger CC Plymate SR . The link between androgen receptor splice variants and castration-resistant prostate cancer. Horm Cancer. (2014) 5:207–17. doi: 10.1007/s12672-014-0177-y 24798453 PMC4308035

[51] Yuan X Cai C Chen S Chen S Yu Z Balk SP . Androgen receptor functions in castration-resistant prostate cancer and mechanisms of resistance to new agents targeting the androgen axis. Oncogene. (2014) 33:2815–25. doi: 10.1038/onc.2013.235 23752196 PMC4890635

[52] Sawant M Mahajan K Renganathan A Weimholt C Luo J Kukshal V . Chronologically modified androgen receptor in recurrent castration-resistant prostate cancer and its therapeutic targeting. Sci Transl Med. (2022) 14:eabg4132. doi: 10.1126/scitranslmed.abg4132 35704598 PMC10259236

[53] Conteduca V Jayaram A Romero-Laorden N Wetterskog D Salvi S Gurioli G . Plasma androgen receptor and docetaxel for metastatic castration-resistant prostate cancer. Eur Urol. (2019) 75:368–73. doi: 10.1016/j.eururo.2018.09.049 30773204 PMC6377278

[54] de Wit R de Bono J Sternberg CN Fizazi K Tombal B Wülfing C . Cabazitaxel versus abiraterone or enzalutamide in metastatic prostate cancer. N Engl J Med. (2019) 381:2506–18. doi: 10.1056/NEJMoa1911206 31566937

[55] Dallos MC Obradovic AZ McCann P Chowdhury N Pratapa A Aggen DH . Androgen deprivation therapy drives a distinct immune phenotype in localized prostate cancer. Clin Cancer Res. (2024) 30:5218–30. doi: 10.1158/1078-0432.CCR-24-0060 39269310 PMC11905119

[56] Pelton K Freeman MR Solomon KR . Cholesterol and prostate cancer. Curr Opin Pharmacol. (2012) 12:751–59. doi: 10.1016/j.coph.2012.07.006 22824430 PMC3515742

[57] Wu X Daniels G Lee P Monaco ME . Lipid metabolism in prostate cancer. Am J Clin Exp Urol. (2014) 2:111–20. 25374912 PMC4219300

[58] Luo Y Yang Z Yu Y Zhang P . HIF1alpha lactylation enhances KIAA1199 transcription to promote angiogenesis and vasculogenic mimicry in prostate cancer. Int J Biol Macromol. (2022) 222:2225–43. doi: 10.1016/j.ijbiomac.2022.10.014 36209908

[59] Galbraith L Leung HY Ahmad I . Lipid pathway deregulation in advanced prostate cancer. Pharmacol Res. (2018) 131:177–184. doi: 10.1016/j.phrs.2018.02.022 29466694

[60] Tucci M Zichi C Buttigliero C Vignani F Scagliotti GV Di Maio M . Enzalutamide-resistant castration-resistant prostate cancer: challenges and solutions. Onco Targets Ther. (2018) 11:7353–68. doi: 10.2147/OTT.S153764 30425524 PMC6204864

[61] Kita Y Goto T Akamatsu S Yamasaki T Inoue T Ogawa O . Castration-resistant prostate cancer refractory to second-generation androgen receptor axis-targeted agents: opportunities and challenges. Cancers (Basel). (2018) 10:345. doi: 10.3390/cancers10100345 30248934 PMC6210307

[62] Coutinho I Day TK Tilley WD Selth LA . Androgen receptor signaling in castration-resistant prostate cancer: a lesson in persistence. Endocr Relat Cancer. (2016) 23:T179–97. doi: 10.1530/ERC-16-0422 27799360

[63] Graham L Schweizer MT . Targeting persistent androgen receptor signaling in castration-resistant prostate cancer. Med Oncol. (2016) 33:44. doi: 10.1007/s12032-016-0759-3 27042852

[64] Zhao F Zhang T Wei J Chen L Liu Z Jin Y . Integrated single-cell transcriptomic analyses identify a novel lineage plasticity-related cancer cell type involved in prostate cancer progression. EBioMedicine. (2024) 109:105398. doi: 10.1016/j.ebiom.2024.105398 39418984 PMC11530610

[65] Wong HY Sheng Q Hesterberg AB Croessmann S Rios BL Giri K . Single cell analysis of cribriform prostate cancer reveals cell intrinsic and tumor microenvironmental pathways of aggressive disease. Nat Commun. (2022) 13:6036. doi: 10.1038/s41467-022-33780-1 36229464 PMC9562361

[66] Chen CC Tran W Song K Sugimoto T Obusan MB Wang L . Temporal evolution reveals bifurcated lineages in aggressive neuroendocrine small cell prostate cancer trans-differentiation. Cancer Cell. (2023) 41:2066–82.e9. doi: 10.1016/j.ccell.2023.10.009 37995683 PMC10878415

[67] Wang Z Wang T Hong D Dong B Wang Y Huang H . Single-cell transcriptional regulation and genetic evolution of neuroendocrine prostate cancer. iScience. (2022) 25:104576. doi: 10.1016/j.isci.2022.104576 35789834 PMC9250006

[68] Zhang T Zhao F Lin Y Liu M Zhou H Cui F . Integrated analysis of single-cell and bulk transcriptomics develops a robust neuroendocrine cell-intrinsic signature to predict prostate cancer progression. Theranostics. (2024) 14:1065–80. doi: 10.7150/thno.92336 38250042 PMC10797290

[69] Gu Y Ly A Rodriguez S Zhang H Kim J Mao Z . PD-1 blockade plus cisplatin-based chemotherapy in patients with small cell/neuroendocrine bladder and prostate cancers. Cell Rep Med. (2024) 5:101824. doi: 10.1016/j.xcrm.2024.101824 39536751 PMC11604497

[70] Jiang C Song Y Rorive S Allard J Tika E Zahedi Z . Innate immunity and the NF-kappaB pathway control prostate stem cell plasticity, reprogramming and tumor initiation. Nat Cancer. (2025) 6:1537–58. doi: 10.1038/s43018-025-00994-3 40550901 PMC7618034

[71] Hong J Bae S Cavinato L Seifert R Ryhiner M Rominger A . Deciphering the effects of radiopharmaceutical therapy in the tumor microenvironment of prostate cancer: an in-silico exploration with spatial transcriptomics. Theranostics. (2024) 14:7122–39. doi: 10.7150/thno.99516 39629134 PMC11610134

[72] Sjöström M Zhao SG Levy S Zhang M Ning Y Shrestha R . The 5-hydroxymethylcytosine landscape of prostate cancer. Cancer Res. (2022) 82:3888–3902. doi: 10.1158/0008-5472.CAN-22-1123 36251389 PMC9627125

[73] Mizuno K Ku SY Venkadakrishnan VB Bakht MK Sigouros M Chan J . Intraindividual epigenetic heterogeneity underlying phenotypic subtypes of advanced prostate cancer. Nat Commun. (2025) 16:5543. doi: 10.1038/s41467-025-60654-z 40593552 PMC12219151

[74] Guo H Vuille JA Wittner BS Lachtara EM Hou Y Lin M . DNA hypomethylation silences anti-tumor immune genes in early prostate cancer and CTCs. Cell. (2023) 186:2765–82.e28. doi: 10.1016/j.cell.2023.05.028 37327786 PMC10436379

[75] Barfeld SJ Itkonen HM Urbanucci A Mills IG . Androgen-regulated metabolism and biosynthesis in prostate cancer. Endocr Relat Cancer. (2014) 21:T57–66. doi: 10.1530/ERC-13-0515 24497572

[76] Lin C Salzillo TC Bader DA Wilkenfeld SR Awad D Pulliam TL . Prostate cancer energetics and biosynthesis. Adv Exp Med Biol. (2019) 1210:185–237. doi: 10.1007/978-3-030-32656-2_10 31900911 PMC8096614

[77] Mutuku SM Spotbeen X Trim PJ Snel MF Butler LM Swinnen JV . Unravelling prostate cancer heterogeneity using spatial approaches to lipidomics and transcriptomics. Cancers (Basel). (2022) 14:1702. doi: 10.3390/cancers14071702 35406474 PMC8997139

[78] Mosquera MJ Kim S Bareja R Fang Z Cai S Pan H . Extracellular matrix in synthetic hydrogel-based prostate cancer organoids regulate therapeutic response to EZH2 and DRD2 inhibitors. Adv Mater. (2022) 34:e2100096. doi: 10.1002/adma.202100096 34676924 PMC8820841

[79] Tian W Li Y Xiao H Cai S Li J Wen B . Omics integration identified CRIP2 as a key mediator of olaparib resistance in prostate cancer. Transl Androl Urol. (2025) 14:2680–96. doi: 10.21037/tau-2025-543 41132344 PMC12541509

[80] Mateo J Carreira S Sandhu S Miranda S Mossop H Perez-Lopez R . DNA-repair defects and olaparib in metastatic prostate cancer. N Engl J Med. (2015) 373:1697–1708. doi: 10.1056/NEJMoa1506859 26510020 PMC5228595

[81] Maly IV Hofmann WA . Fatty acids and calcium regulation in prostate cancer. Nutrients. (2018) 10:788. doi: 10.3390/nu10060788 29921791 PMC6024573

[82] Fujita K Hayashi T Matsushita M Uemura M Nonomura N . Obesity, inflammation, and prostate cancer. J Clin Med. (2019) 8:201. doi: 10.3390/jcm8020201 30736371 PMC6406330

[83] Ou Y Xia C Ye C Liu M Jiang H Zhu Y . Comprehensive scRNA-seq analysis to identify new markers of M2 macrophages for predicting the prognosis of prostate cancer. Ann Med. (2024) 56:2398195. doi: 10.1080/07853890.2024.2398195 39221762 PMC11370685

[84] Heidegger I Fotakis G Offermann A Goveia J Daum S Salcher S . Comprehensive characterization of the prostate tumor microenvironment identifies CXCR4/CXCL12 crosstalk as a novel antiangiogenic therapeutic target in prostate cancer. Mol Cancer. (2022) 21:132. doi: 10.1186/s12943-022-01597-7 35717322 PMC9206324

[85] Dai J Lu Y Roca H Keller JM Zhang J McCauley LK . Immune mediators in the tumor microenvironment of prostate cancer. Chin J Cancer. (2017) 36:29. doi: 10.1186/s40880-017-0198-3 28292326 PMC5351274

[86] Venturini NJ Drake CG . Immunotherapy for prostate cancer. Cold Spring Harb Perspect Med. (2019) 9:a030627. doi: 10.1101/cshperspect.a030627 30201787 PMC6496329

[87] Byrne MHV Anbarasan T Browning L Woodcock DJ . What spatial omics is teaching us about field cancerisation in prostate and bladder cancer. BJU Int. (2025) 136:578–89. doi: 10.1111/bju.16830 40566675 PMC12415322

[88] Bonstingl L Zinnegger M Sallinger K Pankratz K Müller CT Pritz E . Advanced single-cell and spatial analysis with high-multiplex characterization of circulating tumor cells and tumor tissue in prostate cancer: unveiling resistance mechanisms with the CoDuCo in situ assay. biomark Res. (2024) 12:140. doi: 10.1186/s40364-024-00680-z 39550585 PMC11568690

[89] Feng W Ladewig E Lange M Salsabeel N Zhao H Lee YS . ERG-driven prostate cancer initiation is cell-context dependent and requires KMT2A and DOT1L. Nat Genet. (2025) 57:2177–91. doi: 10.1038/s41588-025-02289-w 40858905 PMC12425824

[90] Graham MK Wang R Chikarmane R Abel B Vaghasia A Gupta A . Convergent alterations in the tumor microenvironment of MYC-driven human and murine prostate cancer. Nat Commun. (2024) 15:7414. doi: 10.1038/s41467-024-51450-2 39198404 PMC11358296

[91] Crona DJ Milowsky MI Whang YE . Androgen receptor targeting drugs in castration-resistant prostate cancer and mechanisms of resistance. Clin Pharmacol Ther. (2015) 98:582–9. doi: 10.1002/cpt.256 26331358 PMC4715745

[92] Hurwitz M . Chemotherapy in prostate cancer. Curr Oncol Rep. (2015) 17:44. doi: 10.1007/s11912-015-0468-7 26216506

[93] de Bono J Mateo J Fizazi K Saad F Shore N Sandhu S . Olaparib for metastatic castration-resistant prostate cancer. N Engl J Med. (2020) 382:2091–102. doi: 10.1056/NEJMoa1911440 32343890

[94] Sachpekidis C Alberts I Rominger A Afshar-Oromieh A . PSMA radioligand therapy in prostate cancer: overview, latest advances and remaining challenges. Immunotherapy. (2019) 11:1267–71. doi: 10.2217/imt-2019-0146 31496329

[95] Saad F Clarke NW Oya M Shore N Procopio G Guedes JD . Olaparib plus abiraterone versus placebo plus abiraterone in metastatic castration-resistant prostate cancer (PROpel): final prespecified overall survival results of a randomised, double-blind, phase 3 trial. Lancet Oncol. (2023) 24:1094–108. doi: 10.1016/S1470-2045(23)00382-0 37714168

[96] Chi KN Castro E Attard G Smith MR Sandhu S Efstathiou E . Niraparib and abiraterone acetate plus prednisone in metastatic castration-resistant prostate cancer: final overall survival analysis for the phase 3 MAGNITUDE trial. Eur Urol Oncol. (2025) 8:986–98. doi: 10.1016/j.euo.2025.04.012 40328571

[97] Agarwal N Azad AA Carles J Fay AP Matsubara N Szczylik C . Talazoparib plus enzalutamide in men with metastatic castration-resistant prostate cancer: final overall survival results from the randomised, placebo-controlled, phase 3 TALAPRO-2 trial. Lancet. (2025) 406:447–60. doi: 10.1016/S0140-6736(25)00684-1 40683290

[98] Sartor O de Bono J Chi KN Fizazi K Herrmann K Rahbar K . Lutetium-177-PSMA-617 for metastatic castration-resistant prostate cancer. N Engl J Med. (2021) 385:1091–103. doi: 10.1056/NEJMoa2107322 34161051 PMC8446332

[99] Hofman MS Emmett L Sandhu S Iravani A Joshua AM Goh JC . (177)Lu]Lu-PSMA-617 versus cabazitaxel in patients with metastatic castration-resistant prostate cancer (TheraP): a randomised, open-label, phase 2 trial. Lancet. (2021) 397:797–804. doi: 10.1016/S0140-6736(21)00237-3 33581798

[100] Fizazi K Chi KN Shore ND Herrmann K de Bono JS Castellano D . Final overall survival and safety analyses of the phase III PSMAfore trial of [177Lu]Lu-PSMA-617 versus change of androgen receptor pathway inhibitor in taxane-naive patients with metastatic castration-resistant prostate cancer. Ann Oncol. (2025) 36:1319–30. doi: 10.1016/j.annonc.2025.07.003 40680993 PMC12399216

[101] Rodarte KE Nir Heyman S Guo L Flores L Savage TK Villarreal J . Neuroendocrine differentiation in prostate cancer requires ASCL1. Cancer Res. (2024) 84:3522–37. doi: 10.1158/0008-5472.CAN-24-1388 39264686 PMC11534540

[102] Wen XY Wang RY Yu B Yang Y Yang J Zhang HC . Integrating single-cell and bulk RNA sequencing to predict prognosis and immunotherapy response in prostate cancer. Sci Rep. (2023) 13:15597. doi: 10.1038/s41598-023-42858-9 37730847 PMC10511553

[103] Crocetto F Musone M Chianese S Conforti P Digitale Selvaggio G Caputo VF . Blood and urine-based biomarkers in prostate cancer: Current advances, clinical applications, and future directions. J Liq Biopsy. (2025) 9:100305. doi: 10.1016/j.jlb.2025.100305 40606769 PMC12221370

[104] Drost J Karthaus WR Gao D Driehuis E Sawyers CL Chen Y . Organoid culture systems for prostate epithelial and cancer tissue. Nat Protoc. (2016) 11:347–58. doi: 10.1038/nprot.2016.006 26797458 PMC4793718

[105] Pitzen SP Rudenick AN Qiu Y Zhang W Munro SA McCluskey BM . Comparative transcriptomics reveals a mixed basal, club, and hillock epithelial cell identity in castration-resistant prostate cancer. Proc Natl Acad Sci USA. (2025) 122:e2415308122. doi: 10.1073/pnas.2415308122 39913208 PMC11831193

[106] Schweizer MT Yu EY . Persistent androgen receptor addiction in castration-resistant prostate cancer. J Hematol Oncol. (2015) 8:128. doi: 10.1186/s13045-015-0225-2 26566796 PMC4644296

[107] McCrea E Sissung TM Price DK Chau CH Figg WD . Androgen receptor variation affects prostate cancer progression and drug resistance. Pharmacol Res. (2016) 114:152–62. doi: 10.1016/j.phrs.2016.10.001 27725309 PMC5154811

